# MK-8527 is a novel inhibitor of HIV-1 reverse transcriptase translocation with potential for extended-duration dosing

**DOI:** 10.1371/journal.pbio.3003308

**Published:** 2025-08-26

**Authors:** Izzat T. Raheem, Vinay Girijavallabhan, Kerry L. Fillgrove, Shih Lin Goh, Carolyn Bahnck-Teets, Qian Huang, Fangbiao Li, Bang-Lin Wan, Gregory T. O’Donnell, Jonathan B. Patteson, Maria E. Cilento, Amrith Bennet, Robert P. Hayes, Srivanya Tummala, Carolyn McHale, Judyann Wiltsie, Joan Ellis, Ernest Asante-Appiah, Daria J. Hazuda, Jeffrey Hale, Jay A. Grobler, Min Xu, Tracy L. Diamond, Ming-Tain Lai

**Affiliations:** Merck & Co., Inc., Rahway, New Jersey, United States of America; The University of Texas Medical Branch at Galveston, UNITED STATES OF AMERICA

## Abstract

Nucleoside reverse transcriptase translocation inhibitors (NRTTIs) are potent antiretroviral agents that block HIV replication. A comprehensive lead optimization campaign was undertaken to develop a novel long-acting NRTTI with the potential for extended-duration dosing for HIV prophylaxis. Broad exploration of nucleoside structure–activity relationship (SAR), leveraging ribose core, periphery, and nucleobase modifications, along with systematic progression of compounds of interest through key in vitro and in vivo studies led to the discovery of MK-8527. MK-8527 is a novel deoxyadenosine analog that is phosphorylated intracellularly to its active triphosphate (TP) form, which inhibits reverse transcription. Iron footprinting and primer extension assays demonstrated that MK-8527-TP inhibits translocation of reverse transcriptase on the primer and template, and this inhibition allows for both immediate and delayed chain termination of reverse transcription. MK-8527 inhibits viral replication in human peripheral blood mononuclear cells (PBMCs), with a half maximal inhibitory concentration (IC_50_) of 0.21 nM. The pharmacokinetic (PK) profile of MK-8527 in rats and rhesus monkeys was characterized by low-to-moderate clearance and volume of distribution, with good oral absorption (57% and 100% in rats and monkeys, respectively). Following oral administration of MK-8527 to monkeys, MK-8527-TP exhibited an intracellular half-life of approximately 48 h in PBMCs, significantly longer than the apparent plasma half-life of the parent compound (approximately 7 h). MK-8527 and MK-8527-TP demonstrated favorable in vitro off-target profiles, with IC_50_ values of ≥95 µM against human DNA polymerases tested, and no off-target activities at 10 μM against a panel of 114 enzyme and receptor binding assays. Collectively, the potent antiretroviral activity and favorable preclinical PK and off-target profiles make MK-8527 an attractive clinical candidate, and it is currently in clinical trials for once-monthly oral HIV-1 pre-exposure prophylaxis.

## Abstract 2

Nucleoside reverse transcriptase translocation inhibitors (NRTTIs) are antiretroviral agents that block HIV replication. This study identifies a novel long-acting deoxyadenosine analog, MK-8527, that displays potent antiretroviral activity and favorable preclinical pharmacokinetic and off-target profiles in non-human primate models of HIV-1 infection.

## Introduction

Since the beginning of the HIV pandemic, significant treatment advances have been made for people living with HIV. The advent of combination antiretroviral (ARV) regimens has transformed the outcome of HIV infection from fatal illness to a manageable, chronic condition [1]. Currently, approved ARVs fall into 6 main classes: nucleos(t)ide reverse transcriptase inhibitors (NRTIs), non-nucleoside reverse transcriptase inhibitors (NNRTIs), protease inhibitors, integrase strand transfer inhibitors, entry inhibitors, and capsid inhibitors. To maintain HIV suppression, a treatment regimen containing at least 2 or 3 ARVs that inhibit HIV replication via different mechanisms is required. The lifelong need for daily adherence and the emergence of HIV drug resistance remain ongoing challenges to optimal, persistent viral suppression [2,3].

UNAIDS, the Joint United Nations Programme on HIV/AIDS, has set an ambitious “95/95/95” target for 2025, with the goal of 95% of people living with HIV knowing their HIV status, 95% of people who know they are living with HIV being on treatment, and 95% of those on treatment maintaining suppression of the virus [4]. To achieve these goals, significant research has focused on the development of more tolerable and convenient ARV regimens to decrease barriers associated with adherence and to improve overall treatment outcomes [5]. Additionally, in the absence of HIV vaccines, prophylactic agents are key to decreasing the number of new infections. The most commonly used prophylactic option is daily oral therapy with emtricitabine/tenofovir disoproxil fumarate or emtricitabine/tenofovir alafenamide (FTC/TDF or FTC/TAF), which have shown high effectiveness against HIV acquisition when taken daily, as indicated [6]. However, adherence to daily pre-exposure prophylaxis is negatively influenced by factors such as HIV-related stigma, concerns about adverse events, and a low perceived risk of acquiring HIV [6–9]. Different types of potential long-acting HIV prevention options (i.e., once weekly dosing or longer), including injectables, broadly neutralizing monoclonal antibodies, and intravaginal rings, have been evaluated in clinical trials [6,10–15].

Recently, the twice-yearly injectable HIV-1 capsid inhibitor, lenacapavir, was found to reduce the likelihood of acquiring HIV-1 in cisgender women and men, transgender men, transgender women, and gender non-binary individuals, in two phase 3 trials [12,16]. Based on these results, twice-yearly injectable lenacapavir was approved for HIV pre-exposure prophylaxis by the U.S. Food and Drug Administration (FDA) in June 2025. Previously, the only approved long-acting prophylactic option was cabotegravir, which is administered via intramuscular injection by a healthcare provider once every 8 weeks [17,18]. Acceptance and accessibility of this regimen is limited by the healthcare service delivery system, the frequency of clinic visits, and potential side effects, particularly injection-site reactions [19,20]. As such, while over 6 million people globally had initiated HIV pre-exposure prophylaxis by the end of 2023, there were still an estimated 1.3 million new HIV infections that year [21,22]. Therefore, the need remains for new, more flexible, acceptable, and accessible long-acting options for HIV pre-exposure prophylaxis.

**Fig 1:**
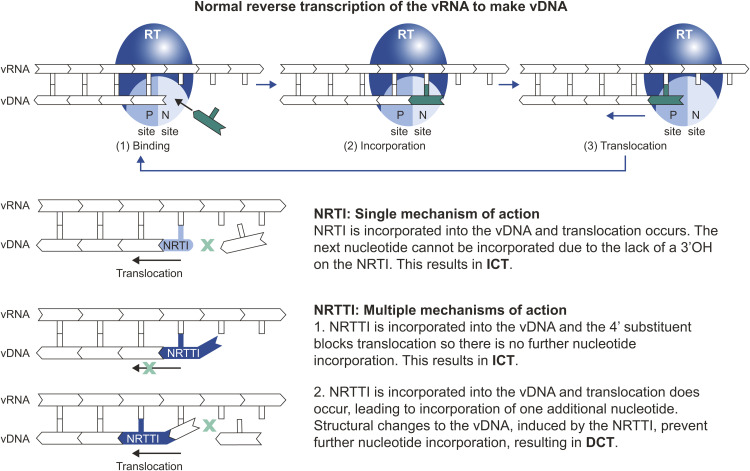
NRTTIs inhibit reverse transcription through multiple unique mechanisms, including inhibition of translocation. DCT, delayed chain termination; ICT, immediate chain termination; NRTI, nucleos(t)ide reverse transcriptase inhibitor; NRTTI, nucleoside reverse transcriptase translocation inhibitor; RT, reverse transcriptase; vDNA, viral DNA, vRNA, viral RNA.

Nucleoside reverse transcriptase translocation inhibitors (NRTTIs) inhibit viral replication by a novel mechanism of action [23–26], and have demonstrated potential as long-acting agents [27–29]. NRTTIs are potent nucleoside analogs that are converted to their pharmacologically active triphosphate (TP) form via endogenous intracellular kinases. As shown in Fig 1, in contrast to all approved NRTIs, NRTTIs maintain a 3′-OH and are not obligate chain terminators. They inhibit reverse transcription by multiple distinct mechanisms. Firstly, the 4′ substituent blocks translocation of reverse transcriptase (RT) along the primer:template, preventing additional deoxynucleotide triphosphate (dNTP) binding and incorporation and resulting in immediate chain termination (ICT). Secondly, in the event that translocation does occur, the 3′-OH allows for the addition of one extra nucleotide before structural changes or distortion in the viral DNA occur, preventing further incorporation; this results in delayed chain termination (DCT) [23,24,30,31]. The mechanism resulting in DCT protects NRTTI-terminated primers from excision, thereby reducing the number of potential mechanisms of resistance to NRTTIs compared with approved NRTIs [23].

To our knowledge, islatravir (ISL) is the first NRTTI to be evaluated in the clinic, revealing the potential for long dosing intervals at low doses. Oral ISL is under investigation in Phase 3 clinical trials for daily and weekly treatment of HIV-1 in combination with other ARVs [32–34]. This work describes the discovery and mechanistic investigation of a novel NRTTI, MK-8527, that was developed through a comprehensive lead optimization campaign focused on identifying structurally and functionally novel NRTTIs, with potential for extended-duration dosing for HIV pre-exposure prophylaxis.

## Results

### Discovery of MK-8527

**Fig 2:**
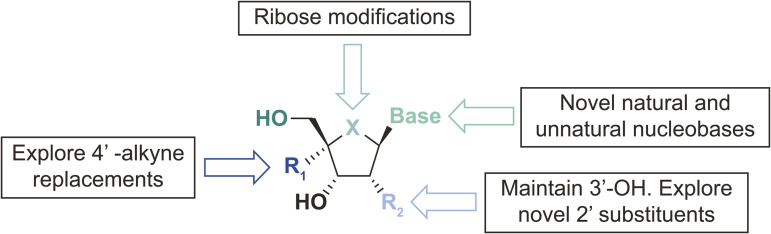
Medicinal chemistry strategy and overview of expansive SAR campaign. Modifications evaluated changes to the periphery of the ribose core, including 4′, 2′, and nucleobase positions, as well as replacement of the ribose core itself. SAR, structure–activity relationship.

**Fig 3:**
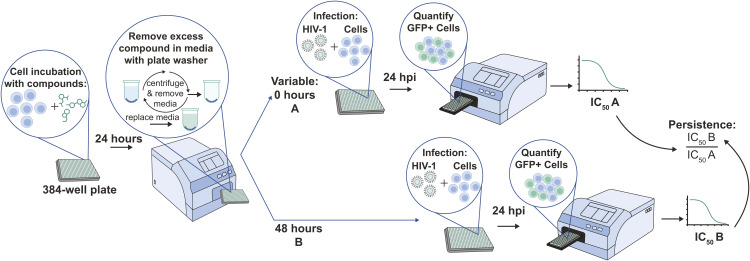
In vitro persistence assay to assess maintenance of effective compound levels after washout. Cells were incubated with test compound for 24 h, allowing time for uptake and phosphorylation, followed by washout. Infection was performed immediately after washout **(A)** or 48 h later **(B)**. Plates were analyzed 24 h post-infection for the number of GFP–positive cells. IC_50_ values were determined, and a persistence ratio was calculated for each compound. GFP, green fluorescent protein; hpi, hours post-infection; IC_50_, half maximal inhibitory concentration.

Discovery efforts toward novel NRTTIs leveraged long-standing experience with ISL, which served as a robust starting point. Medicinal chemistry approaches initially focused on broad evaluation of the nucleoside periphery with single-point modifications that used both traditional and novel structural diversity (Fig 2). This effort was then expanded upon by broader structure–activity relationship (SAR) exploration. Preliminary compound profiling focused on evaluation of the antiviral activity of novel nucleosides against wild-type (WT) HIV-1 in a multicycle antiviral assay in MT4-GFP (MT4 cells engineered to express green fluorescent protein [GFP]) cells and in a single-cycle assay in peripheral blood mononuclear cells (PBMCs), as well as assessment of cellular cytotoxicity. Persistence of antiviral activity was also evaluated in MT4-GFP cells and human PBMCs to understand whether compound levels sufficient for antiviral activity could be maintained in cells after washout (in which lower persistence values indicated compound persistence after washout; see Fig 3).

Given previously described SAR [35–37], a well-understood mechanism of action, and knowledge of how ISL-triphosphate (ISL-TP) binds to the RT catalytic active site [24–26,30,31], limited flexibility in the structural modifications that were well tolerated was anticipated. Indeed, comprehensive efforts to substitute the 4′-ethynyl group or further modify the 2′, 3′, or 4′ positions of the ribose core did not lead to identification of compounds with improved profiles. Because the 4′ alkyne occupies a small and critical hydrophobic pocket in the catalytic active site [26], all efforts to substitute the alkyne terminus (i.e., methyl, aryl, carboxyl) were met with significant losses in antiviral potency. Although small isosteric alkyne replacements such as CN, methyl, and azide maintained measurable potency as previously reported [37], other more significant changes (i.e., aryl, halomethyl, azidomethyl, methoxymethyl, and carboxamide) resulted in near-complete loss of activity. Similarly, changes made to the 3′ and 2′ positions resulted in generally inactive compounds.

**Fig 4:**
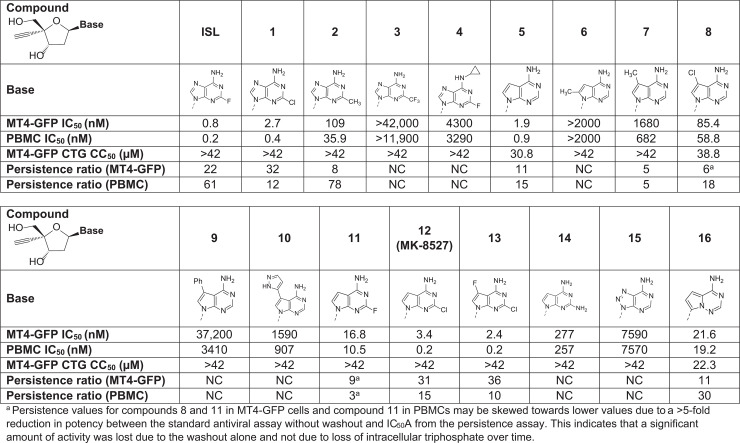
Evaluation of novel nucleobase substitutions. Potency, cytotoxicity, and persistence ratios in MT4-GFP cells and/or PBMCs for ISL and 16 compounds with nucleobase substitutions. CC_50_, concentration of test compound required to reduce cell viability by 50%; CTG, CellTiter-Glo; GFP, green fluorescent protein; IC_50_, half maximal inhibitory effect; ISL, islatravir; NC, not calculated (i.e., persistence ratios could not be calculated due to qualifiers in IC_50_A [infection performed immediately after washout] and/or IC_50_B [infection performed 48 h after washout]); PBMC, peripheral blood mononuclear cell.

Despite the lack of promising compounds identified from evaluation of the 2′, 3′, and 4′ positions of the nucleoside, investigation of the nucleobase structure and the ribose core itself proved more productive. Fig 4 includes representative nucleobase modifications, and highlights the most productive path of discovery, which resulted in the identification of the lead candidate, MK-8527. Most of the nucleobase SAR was anchored around purine analogs and isosteres. Although relatively minor structural changes at the 2-position were tolerated (Fig 4, Compound 1), larger aliphatic and aromatic substitution as well as significant changes in the nucleobase electron density (Fig 4, Compounds 2–3) resulted in partial or complete loss of antiviral activity, as did aliphatic substitution of the 2-amino group (Fig 4, Compound 4). Despite the highlighted structural and electronic sensitivities of the nucleobase, modification to a 7-deazaadenine group was well tolerated, maintained good antiviral potency, and showed reduced cytotoxicity (as measured by the CellTiter-Glo [CTG] assay) profiles, with reasonable in vitro persistence (Fig 4, Compound 5). However, like its adenine counterparts, this nucleobase family was sensitive to substitution, with any sizeable modification at the 2-, 7-, or 8-position resulting in loss of antiviral activity (Fig 4, Compounds 6–10). Small substitutions remained tolerated, and 2-chloro-7-deazaadenine proved to be an optimal nucleobase with respect to antiviral potency, cytotoxicity, and persistence compared with the corresponding 2-fluoro and 2-amino analogs (Fig 4, Compounds 11–14). Compounds 12 and 13, in particular, exhibited single-digit nanomolar potency in the MT4-GFP cell line and subnanomolar potency in human PBMCs. Further exploration of the 7-deazaadenine core was unproductive. Additionally, a triazolopyrimidine (Fig 4, Compound 15) was completely inactive, while *C*-linked adenine isosteres provided modest potency but exhibited compromised cytotoxicity and persistence profiles compared with other leads (Fig 4, Compound 16).

**Fig 5:**
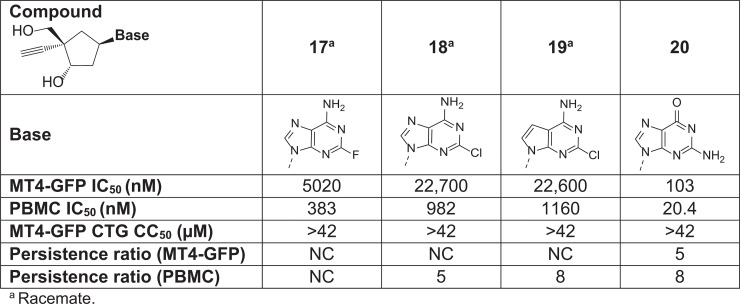
Evaluation of carbaribose modifications. Potency, cytotoxicity, and persistence ratios in MT4-GFP cells and/or PBMCs for four compounds with carbaribose modifications. CH_2_, methylene; CC_50_, concentration of test compound required to reduce cell viability by 50%; CTG, CellTiter-Glo; GFP, green fluorescent protein; IC_50_, half maximal inhibitory effect; NC, not calculated (i.e., persistence ratios could not be calculated due to qualifiers in IC_50_A [infection performed immediately after washout] and/or IC_50_B [infection performed 48 h after washout]); PBMC, peripheral blood mononuclear cell.

With a limited number of novel nucleobases of interest identified, focus was changed to an evaluation of modifications to the ribose core. Several key structural changes were initially targeted, including aza-, thio-, and carbaribose. Of these, the corresponding methylene (CH_2_) carbaribose was initially the most compelling but, while synthetically accessible, the compound was less potent compared with its ribose counterpart. As previously described [38], further exploration of the SAR of this unique nucleoside ring system revealed that concomitant optimization of the nucleobase (Fig 5, Compounds 17–20) and subsequent use of a prodrug strategy significantly improved overall potency while delivering compounds that also had favorable overall pharmacokinetics (PK) profiles.

Through the efforts described above, Compounds 12 and 13 emerged as lead candidates, and expanded evaluation of both highlighted a superior overall profile for Compound 12 over Compound 13, particularly with respect to efficiency of intracellular TP formation*.* Compound 12 (MK-8527) was thus selected as a novel and differentiated candidate with the appropriate balance of preclinical in vitro, in vivo, and physicochemical properties for continued progression as a long-acting NRTTI, with the potential for extended-duration dosing; details are described in later sections of this manuscript.

### MK-8527 is a potent inhibitor of HIV replication

The in vitro antiretroviral profile of MK-8527 is presented in Table 1. MK-8527 potently inhibited viral replication in PBMCs with an IC_50_ of 0.21 nM. The concentration of intracellular MK-8527-TP required for antiviral activity in PBMCs incubated with MK-8527 for 24 h was determined by liquid chromatography tandem mass spectrometry (LC–MS/MS). However, in PBMCs incubated with MK-8527 at its antiviral IC_50_ concentration (0.21 nM), MK-8527-TP was below the limit of quantitation. As a linear relationship was observed between the concentration of MK-8527 added to the incubation and the determined intracellular MK-8527-TP concentrations, the intracellular concentration of MK-8527-TP corresponding to 0.21 nM MK-8527 (i.e., the antiviral IC_50_) was extrapolated from three experiments (each performed in triplicate) and determined to be 0.0092 ± 0.0035 pmol per 10^6^ cells.

**Table 1:** Antiviral activity of MK-8527 and ISL in multiple assays and cell types.

			MK-8527	ISL
Antiviral potency as IC_50_, nM	HIV-1 – PBMCs		0.21 ± 0.09 (*n* = 93)	0.23 ± 0.04 (*n* = 4)^a^
	HIV-1 – MT4-GFP cells		3.37 ± 1.00 (*n* = 33)	0.80 ± 0.08 (*n* = 11)^a^
	HIV-1 – CEM-SS cells		0.14	N/A
	HIV-2 – CEM-SS cells		0.007	N/A
Persistence assay	MT4-GFP cells	IC_50_A	7.2 ± 2.3 nM (*n* = 11)	0.89 ± 0.63 nM (*n* = 36)
		IC_50_B	223.2 ± 103.8 nM (*n* = 10)	25.5 ± 9.96 nM (*n* = 6)
		Persistence ratio	31	22
	PBMCs	IC_50_A	1.2 ± 0.2 nM (*n* = 7)	0.8 ± 0.48 nM (*n* = 7)
		IC_50_B	18.1 ± 4.8 nM (*n* = 9)	39.7 ± 23.9 nM (*n* = 12)
		Persistence ratio	15	61

IC_50_, half maximal inhibitory concentration; GFP, green fluorescent protein; N/A, data not available; PBMC, peripheral blood mononuclear cell.

IC_50_A denotes that infection was performed immediately after washout; IC_50_B denotes that infection was performed 48 h after washout.

Potency values are presented as IC_50_ ± standard deviation when data from ≥3 replicates were available.

^a^Data from Diamond, and colleagues [39].

**Fig 6:**
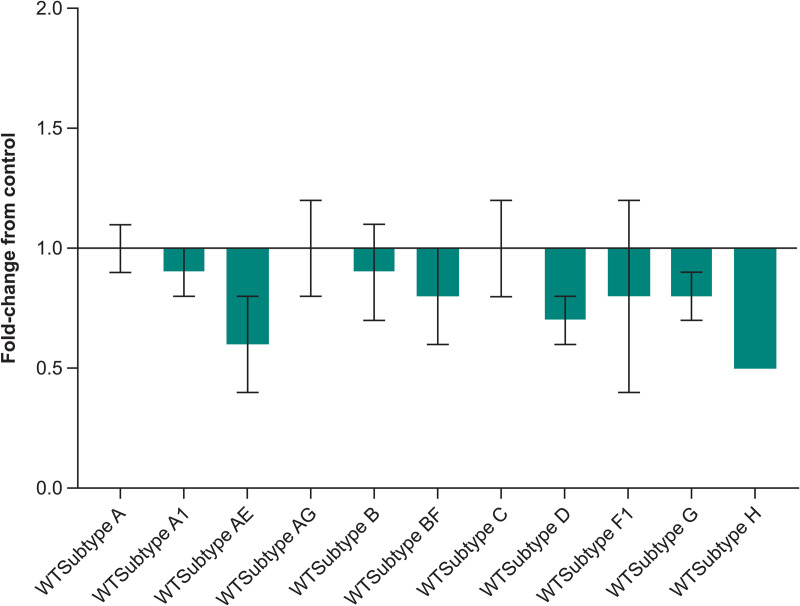
Activity of MK-8527 against HIV-1 subtypes. Antiviral activity of MK-8527 was evaluated across 11 HIV-1 subtypes with the PhenoSense assay using vectors encoding protease and RT sequences derived from HIV-1 in human plasma for WT subtypes A (*n* = 3), A1 (*n* = 5), AE (*n* = 5), AG (*n* = 6), B (*n* = 5), BF (*n* = 5), C (*n* = 5), D (*n* = 4), F1 (*n* = 5), and G (*n* = 2). Geometric mean fold change from CNDO control is shown; error bars denote standard deviation. The underlying data for Fig 6 can be found in the S1 Data. RT, reverse transcriptase; WT, wild-type.

In addition to the antiviral activity of MK-8527 in PBMCs, its potency was also evaluated against HIV-1 in MT4-GFP cells and HIV-1 and HIV-2 in CEM-SS cells, with a consistent low nanomolar to subnanomolar potency observed in all assays. MK-8527 also demonstrated consistent potency across HIV-1 subtypes in the PhenoSense assay (Monogram Biosciences; South San Francisco, CA, USA) [40] conducted in HEK293T cells (Fig 6).

As described previously, an assay was also performed to determine the persistence of MK-8527 activity after washout in MT4-GFP cells and human PBMCs (Fig 3 and Table 1). Compounds were incubated with cells for 24 h and then washed off; infection was performed immediately after washout (IC_50_A) or 48 h after washout (IC_50_B). Similar to the previous assays, MK-8527 had an IC_50_A with low nM potency; however, as previously shown (Fig 5), the potency was decreased with IC_50_B leading to persistence ratios of 31- and 15-fold in MT4-GFP cells and PBMCs, respectively, likely due to a loss in MK-8527-TP in the cells over time after washout.

### MK-8527-TP directly inhibits HIV-1 RT

To verify that the antiviral effects of MK-8527 were a result of direct inhibition of HIV-1 RT, MK-8527-TP was evaluated in a biochemical assay using purified recombinant RT. MK-8527-TP inhibited RNA-dependent DNA polymerization, with an IC_50_ of 813 ± 238 nM (*n* = 8). This is comparable to what was previously reported for ISL-TP in a similar assay (263 nM) [38].

### MK-8527 is a specific inhibitor of HIV

In order to determine whether MK-8527 activity was specific for HIV, activity of MK-8527 was evaluated against a panel of unrelated viruses, including *Hepadnaviridae* (hepatitis B virus), *Flaviviridae* (hepatitis C virus, dengue virus, West Nile virus), *Adenoviridae* (adenovirus-5), *Arenaviridae* (Tacaribe virus), *Bunyaviridae* (Rift Valley fever virus), *Caliciviridae* (murine norovirus), *Herpesviridae* (herpes simplex virus-1 and -2), *Orthomyxoviridae* (influenza A and B virus), *Pneumoviridae* (respiratory syncytial virus), *Picornaviridae* (poliovirus-1 and enterovirus 68), and *Togaviridae* (Venezuelan equine encephalitis virus). MK-8527 did not display antiviral activity or cytotoxicity up to the highest concentration tested (20 µM for hepatitis C virus and 50 µM for all other viruses) in any of these assays.

### MK-8527 has a favorable off-target profile in vitro

MK-8527 and MK-8527-TP were tested at 10 µM in a panel of 114 enzyme and receptor-binding assays consisting of 70 G-protein coupled receptor, 13 ion channel, 7 kinase, 7 nuclear hormone receptor, 6 transporter, 4 protease, 2 neurotransmitter metabolism, 2 lipid metabolism, 2 oxidoreductase, and 1 phosphodiesterase assays. No off-target activity was detected; observed IC_50_ values were >10 µM in all assays.

Mechanistically, the potential for MK-8527-TP to inhibit similar enzymes was evaluated by assessing the inhibitory activity of MK-8527-TP against human DNA polymerases α and β and human mitochondrial DNA polymerase γ. MK-8527-TP showed no inhibitory activity against human DNA polymerase β or human mitochondrial DNA polymerase γ at the highest concentration tested (200 µM), and very weak inhibition of human DNA polymerase α (IC_50_ = 95 µM).

### MK-8527-TP inhibits RT through inhibition of translocation

**Fig 7:**
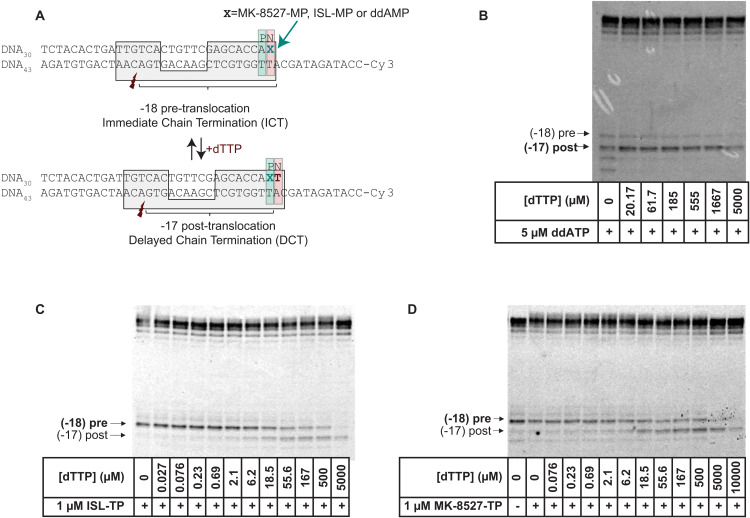
Iron footprinting assay [24] to evaluate the position of RT on the primer and template. An iron footprinting assay **(A)** was performed in the presence of **(B)** 5 µM ddATP; **(C)** 1 µM ISL-TP; or **(D)** µM MK-8527-TP; and escalating concentrations of dTTP. The original uncropped blots can be found in S1 Raw Images. DCT, delayed chain termination; ddATP, dideoxyadenosine triphosphate; dTTP, deoxythymidine triphosphate; ICT, immediate chain termination; ISL, islatravir; MP, monophosphate; RT, reverse transcriptase; TP, triphosphate.

To determine if MK-8527-TP inhibits RT by blocking translocation, an iron footprinting assay was performed as described in Fig 7A. The assay assessed whether RT in the MK-8527-monophosphate/primer:template/RT complex was positioned in a pre-translocation or post-translocation state (i.e., whether MK-8527 blocks reverse transcription at the dNTP-binding site (N-site) or the post-translocation P-site). Cleavage of the template at −18 indicated that the RT was in a pre-translocation state; cleavage of the template at −17 generated a slightly shorter fragment that indicated the RT was in a post-translocation state. In the presence of dideoxyadenosine triphosphate (ddATP), an NRTI that acts as an obligate chain terminator, the RT was mostly in the post-translocation state regardless of the absence or presence of the next nucleotide, deoxythymidine triphosphate (dTTP) (Fig 7B). This observation indicated that translocation occurred once ddATP was incorporated onto the primer and template as ddAMP, whether the next nucleotide was present or not. This finding was different from what was observed for ISL-TP and MK-8527-TP (Fig 7C and 7D). In the presence of ISL-TP and MK-8527-TP, HIV RT remained in a pre-translocation (−18) state in the absence of dTTP, and post-translocation state products (−17) only began accumulating once dTTP concentrations were greater than approximately 6.2 µM. These findings agree with previous reports on ISL-TP and ddATP and demonstrate that, like ISL-TP, MK-8527-TP acts as a translocation inhibitor [23,24].

### Incorporation of MK-8527-TP results in both ICT and DCT

**Fig 8:**
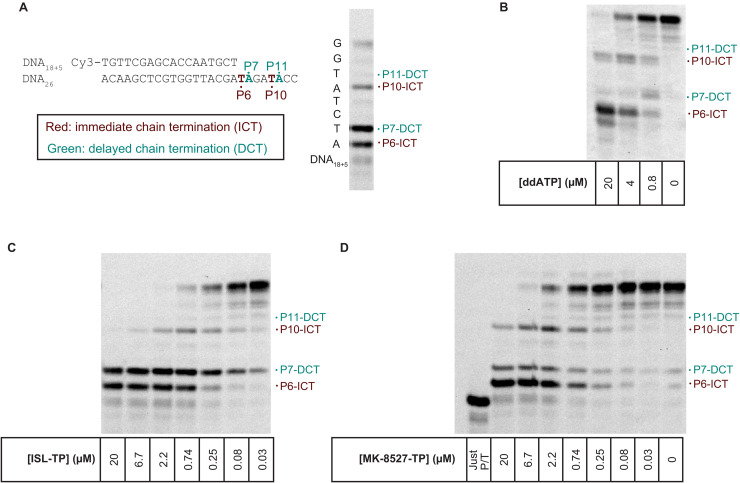
HIV-RT primer extension assay [23] to evaluate the chain termination mechanism of MK-8527-TP compared with ISL-TP and ddATP (an NRTI). **(A)** Primer and template used along with representative gel with DCT and ICT sites marked. Dose-dependent chain termination patterns of **(B)** ddATP, **(C)** ISL-TP, and **(D)** MK-8527-TP are shown. The original uncropped blots can be found in S2 Raw Images. DCT, delayed chain termination; ddATP, dideoxyadenosine triphosphate; ICT, immediate chain termination; ISL-TP, islatravir-triphosphate; NRTI; nucleos(t)ide reverse transcriptase inhibitor, RT, reverse transcriptase; TP, triphosphate.

As shown in Fig 8A, a primer extension assay was performed to assess whether incorporation of MK-8527-TP resulted in ICT and/or DCT. DCT would indicate that translocation could occur, and chain termination occurred after incorporation of the next nucleotide. Reactions included a mixture of the 4 canonical dNTPs (1 µM each) so that extension could continue across the primer template with decreasing concentrations of MK-8527-TP, ISL-TP, or ddATP. The intention was to observe whether extension resulted in accumulation of product due to ICT at position 6 (P6) or 10 (P10), or due to DCT at position 7 (P7) or 11 (P11). When assays were performed in the presence of ddATP (Fig 8B), product accumulated at P6 and P10 only, indicating that ddATP only inhibits via ICT. When assays were performed in the presence of ISL-TP (Fig 8C) or MK-8527-TP (Fig 8D), accumulation of product at P6, P7, and P10 was evident, indicating that both ICT and DCT occurred. In conclusion, the primer extension assays indicated that MK-8527-TP and ISL-TP modulate chain termination by both ICT and DCT. In contrast, ddATP only modulates chain termination by ICT.

### Structure of HIV-RT/DNA in complex with MK-8527-TP

**Fig 9:**
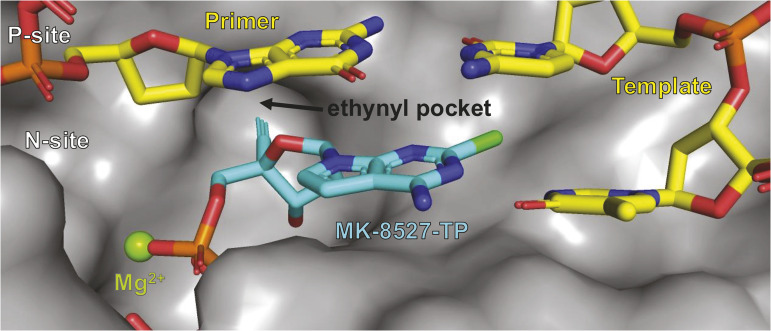
Crystal structure of MK-8527-TP bound in the N-site of a HIV-RT/DNA complex. RT, reverse transcriptase; TP, triphosphate.

To further demonstrate that MK-8527-TP binds at the N-site with the 4′-alkyne group positioned to block translocation, the ternary complex crystal structure of MK-8527-TP with HIV-RT/DNA in the pre-catalytic/pre-translocation states was determined (see S1 Text). The resulting structure, shown in Fig 9, illustrated that MK-8527-TP occupies the N-site and forms the expected noncovalent, base pairing, and magnesium coordination interactions required for catalysis. More importantly, the structure also highlighted projection of the 4′-alkyne group into a key hydrophobic pocket that would sterically hinder translocation by the RT enzyme. These structural observations were consistent with results of the biochemical studies described above and further corroborated the ability of MK-8527-TP to act as a translocation inhibitor. The crystal structure of MK-8727-TP bound in the N-site of HIV-RT/DNA has been deposited in the RCSB protein data bank (pbd code 9DM9).

### PK profile of MK-8527

The PK profile of MK-8527 was evaluated preclinically following intravenous (IV) dosing in Wistar–Hannover rats and rhesus monkeys; the results are summarized in Table 2.

**Table 2:** Pharmacokinetics of MK-8527 in plasma from rats and monkeys.

	IV dose (mg/kg)	CL_p_ (mL/min/kg)	*V*_dss_ (L/kg)	*t*_½_ (hours)	*F* (%)
Rat	1.0	18.1 ± 3.3	2.0 ± 0.3	2.3 ± 1.3	57
Monkey	0.5	12.6 ± 3.3	4.4 ± 0.8	6.7 ± 1.6	100

CL_P_, plasma clearance; *F*, oral bioavailability; IV, intravenous; *t*_½_, half-life; *V*_dss_, volume of distribution at steady state.

**Fig 10:**
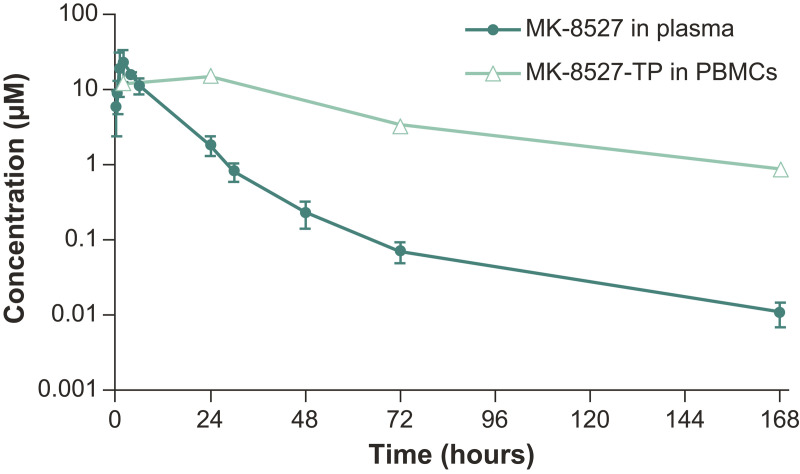
Concentration-vs-time profiles of MK-8527 in plasma and MK-8527-TP in PBMCs following oral administration to rhesus monkeys at 50 mg/kg. The underlying data for Fig 10 can be found in the S2 Data. PBMC, peripheral blood mononuclear cells; TP, triphosphate.

The plasma clearance was relatively low in rats and moderate in monkeys, with a moderate volume of distribution in both species. The plasma half-life was 2.3 and 6.7 h in rats and monkeys, respectively. Following oral administration, the bioavailability of MK-8527 was 57% and 100% in rats and monkeys, respectively. In addition to evaluating plasma PK of MK-8527, the concentration of MK-8527-TP in PBMCs was monitored over time in monkeys following oral administration at 50 mg/kg (Fig 10). The intracellular half-life of MK-8527-TP in PBMCs was 48 h (SD, 11 h), with a similar terminal half-life of MK-8527 in plasma, suggesting that the terminal plasma half-life is partly dependent on the disposition of intracellular MK-8527-TP. Of note, the plasma half-life observed for MK-8527 in the 50 mg/kg oral study is significantly longer than that determined in the intravenous (IV) study, likely due to the terminal phase not being captured well in the IV studies because of limitations in the sensitivity of the assay. Based on allometric scaling from preclinical data, the predicted human PK parameters of MK-8527 were suitable for once-weekly or possibly once-monthly dosing and warranted further evaluation of MK-8527 in the clinic.

## Discussion

The development of combination ARV therapy with effective, tolerable, and convenient dosing regimens, has led to tremendous reductions in HIV-associated morbidity and mortality, allowing HIV to become a manageable, chronic disease [1]. Nonetheless, unmet needs remain for prevention of new HIV infections, globally. Of the currently available ARVs, the mechanisms of action of NRTTIs have emerged as particularly compelling due to their potent antiviral activity and potential for long-acting HIV pre-exposure prophylaxis [27–29,41,42]. Long-acting oral HIV pre-exposure prophylaxis (e.g., once-monthly) could provide an opportunity to address challenges, such as improving the persistence and adherence, compared with a daily pre-exposure prophylaxis regimen.

Herein, we have described the discovery of a novel NRTTI, MK-8527, after thorough SAR evaluation based on ISL scaffold. Comprehensive efforts to modify the 4′-ethynyl group or substitute the 3′ or 2′ positions of the ribose core generally resulted in inactive compounds, whereas modifications to the nucleobase and ribose core were tolerated and provided leads with low nanomolar antiviral activity and favorable cytotoxicity and persistence profiles. Through a rigorous medicinal chemistry campaign, MK-8527 was identified as having an optimal overall profile based on in-depth in vitro and in vivo preclinical studies.

MK-8527 potently inhibited HIV replication, with an IC_50_ of 0.21 nM against HIV-1 in PBMCs, similar to the previously reported IC_50_ of ISL against WT virus in PBMCs (0.23 nM) [39]. The intracellular concentration of TP in PBMCs at the IC_50_ for MK-8527 (0.0092 pmol per 10^6^ cells) is also similar to ISL (0.0097 pmol per 10^6^ cells) [43]. MK-8527 also exhibited potent activity across HIV-1 subtypes and against HIV-2. Furthermore, MK-8527-TP is a specific inhibitor of HIV replication as evidenced by its lack of activity against a diverse panel of viruses in addition to the minimal off-target activities observed against the human DNA polymerases α and β and human mitochondrial DNA polymerase γ (IC_50_ values of ≥95 µM). These activities are not expected to be relevant at clinical exposures.

MK-8527 was demonstrated to act as an NRTTI, whereby it inhibits translocation (Fig 7) leading to ICT and can allow for the incorporation of another nucleoside at high doses, resulting in DCT (Fig 8). The 4′ ethynyl is also positioned in the same hydrophobic pocket (Fig 9) previously reported to be associated with the NRTTI mechanism for ISL [26] and, as described above, has the ability to cause DCT, which protects MK-8527-TP from excision. The presence of a 3′-OH in NRTTIs not only affects the mechanism of reverse transcription inhibition but is also thought to contribute to the potent antiretroviral activity by making NRTTIs better substrates for nucleoside diphosphate kinases, thereby allowing for more efficient phosphorylation of NRTTIs to their active TP, compared with typical NRTIs that lack the 3′-OH [24].

The PK profile of MK-8527 in rats and monkeys was characterized by low-to-moderate clearance and volume of distribution, with good oral absorption. Following oral administration of MK-8527 to monkeys at 50 mg/kg, the half-life of MK-8527-TP in PBMCs was determined to be approximately 48 h. The longer intracellular half-life of the triphosphate compared to the plasma half-life of the parent is consistent with that observed for other nucleoside analogs [44]. The terminal phase of the MK-8527 plasma concentration-vs-time profile was parallel to that of MK-8527-TP in PBMCs and indicated that an equilibrium had been established between MK-8527-TP in PBMCs and MK-8527 in plasma. Once this equilibrium has been established, the plasma concentration of MK-8527 at later timepoints reflect the conversion of MK-8527-TP back to parent drug and the subsequent excretion of MK-8527 from the cell. In this study, the plasma half-life of MK-8527 following oral administration was significantly longer than the plasma half-life observed in the IV dosed study (approximately 7 h). The observed differences in MK-8527 plasma half-life between the 0.5 mg/kg IV dose and the 50 mg/kg oral dose are likely attributed to the inability to fully capture the terminal phase of the concentration versus time profile following IV administration due to concentrations at later time points being below the lower limit of quantification.

Altogether, the potent ARV activity, favorable off-target profile, and PK characteristics suitable for long-acting oral dosing make MK-8527 an attractive clinical candidate. Based on these results and those from phase 1 studies [45,46], further investigation of the potential of MK-8527 as an oral, monthly medication for the prevention of HIV-1 is ongoing in a Phase 2 clinical trial to assess its safety, tolerability and PK when given orally, once monthly to participants at low risk of HIV exposure. Additionally, proof-of-concept studies in non-human primates are ongoing [47]. Monthly oral pre-exposure prophylaxis could provide a convenient option with the potential to improve adherence compared to daily HIV pre-exposure prophylaxis, and to reduce the barrier to access represented by frequent healthcare visits for administration of long-acting injectable HIV pre-exposure prophylaxis.

## Materials and Methods

### Chemistry

**Fig 11:**
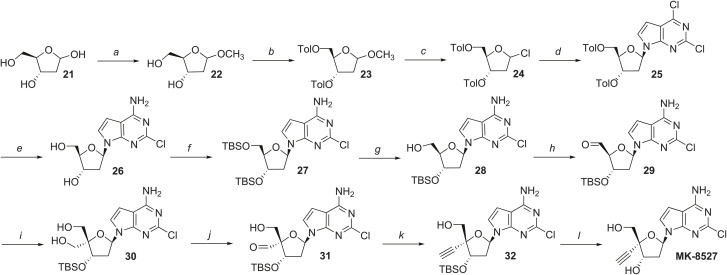
Synthesis of MK-8527. Reagents and conditions: **(a)** acetyl chloride, methanol (MeOH); **(b)** p-toluoyl chloride, pyridine; (c) HCl, diethyl ether; (d) 2,6-dichloro-7-deazapurine, NaH, acetonitrile (MeCN); **(e)** ammonia in isopropanol, sodium methoxide (NaOMe) in methanol (MeOH); (f) TBSCl, imidazole, N,N-dimethylformamide; **(g)** trifluoroacetic acid (TFA), tetrahydrofuran (THF), H2O; (h) IBX, MeCN, dimethyl sulfoxide (DMSO); **(i)** 1. formaldehyde, NaOH, 2. NaBH4, ethanol; **(j)** IBX, MeCN; **(k)** Bestmann−Ohira reagent, K_2_CO_3_; **(l)** TBAF, THF.

An overview of the synthesis of MK-8527 (Compound 12, (2R,3S,5R)-5-(4-amino-2-chloro-7H-pyrrolo2,3-d]pyrimidin-7-yl)- 2-ethynyl-2-(hydroxymethyl)tetrahydrofuran-3-ol)) is shown in Fig 11, beginning with (4*S*,5*R*)-5-(hydroxymethyl)-tetrahydrofuran-2,4-diol (Compound 21). Briefly, Compound 21 was first converted to the corresponding (2*R*,3*S*)-2-(hydroxymethyl)-5-methoxytetrahydrofuran-3-ol (Compound 22) under acidic conditions and then bis-protected with *p-*toluoyl chloride to form Compound 23. Compound 23 was converted to the corresponding 1′-chloride (Compound 24) by treatment with hydrogen chloride (HCl) gas, followed by installation of the 2,4-dichloro-7-deazapurine under strongly basic conditions to provide intermediate nucleoside Compound 25. Protecting group removal with ammonia resulted in concomitant amination of the nucleobase to form Compound 26, which was bis-protected with *tert*-butyldimethylsilyl chloride (TBSCl) and then selectively deprotected at 5′ to furnish 3′-O-*tert*-butyldimethylsilyl analog Compound 28. Oxidation with 2-iodoxybenzoic acid (IBX) to the corresponding aldehyde, and stepwise installation of a 4′-hydroxmethyl group with formaldehyde, and reduction with NaBH_4_ provided intermediate diol compound 30. Selective oxidation, again with IBX, followed by treatment with the Bestmann–Ohira reagent under basic conditions, gave 4′-alkyne analog compound 32. Finally, Compound 32 was deprotected under tetrabutylammonium fluoride (TBAF) conditions to furnish MK-8527 in a 0.5% overall yield over a 12-step sequence. Detailed experimental procedures for the synthesis of MK-8527 and other compounds presented in this manuscript can be found in the S1 Text.

### Multicycle antiviral assay in MT4-GFP cells

Compounds were evaluated for their ability to prevent HIV-1 infection in MT4-GFP cells as described in Diamond, and colleagues [39]. HIV-1 replication was monitored using the MT4-gag-GFP clone D3, and MT4 cells with a GFP reporter gene, the expression of which is dependent on the HIV-1 expressed proteins tat and rev [48]. Infection of MT4-GFP cells with HIV-1 resulted in GFP expression approximately 24 h post-infection. Proviral variants used for these studies were generated by site-directed mutagenesis in the WT R8 proviral plasmid [49]. Virus was produced by transfection in 293T cells.

Briefly, MT4-GFP cells were cultured overnight with the appropriate HIV-1 variant at an approximate multiplicity of infection of 0.01. After approximately 19 h of infection, the cells were washed and resuspended in assay media containing 10% normal human serum (NHS) before being added to 384-well compound plates containing 10-point serial 3-fold dilutions of compound. Controls included no inhibitor (dimethyl sulfoxide [DMSO] only) and a combination of 3 antiviral agents, efavirenz, indinavir, and an integrase strand transfer inhibitor, L-002254051, at final concentrations of 4 µM each. The infected or treated cells were cultured at 37 °C, 5% CO_2_, and 90% humidity until evaluation of infection was conducted at approximately 48 and 72 h post-infection by counting the number of GFP cells in each well using an Acumen eX3 scanner (SPT Labtech; Melbourn, UK). The reproductive ratio, R, was calculated by dividing the number of infected cells at 72 h by the number at 48 h post-infection. The percentage inhibition caused by a test compound was calculated by the following formula:


$$\;inhibition\;=\;[1-(R_{test\;compound}\;-\;R_{positive\;control})]/(R_{DMSO\;only}\;-\;R_{positive\;control})\;\times \;100\%\mathrm{\backslash ]}$$

The dose–response curves of each test compound were plotted as percentage inhibition versus the test concentration. IC_50_ values were determined by nonlinear 4-parameter curve fitting of the dose–response curve data.

### HIV-1 RT biochemical assay

Full-length WT RT protein was expressed in *Escherichia coli* BL21(DE3) cells and purified as described previously [50]. The electrochemiluminescence (ECL) RT biochemical assay was performed based on the previously described protocol [51].

Briefly, the heterodimeric nucleic acid substrate used in the HIV-1 RT polymerase reactions was generated by annealing biotinylated DNA primer to a 500-nucleotide RNA template. The HIV-1 RT enzyme (final concentration of 50 pM) was combined with an inhibitor or DMSO (10% DMSO in the final reaction mixture) in assay buffer containing 62.5 mM Tris-HCl, pH 7.8, 1.25 mM dithiothreitol, 7.5 mM MgCl_2_, 100 mM KCl, 0.03% 3-[(3-cholamidopropyl)-dimethylammonio]-1-propanesulfonate, and 0.125 mM ethylene glycol tetraacetic acid). This mixture was preincubated for 30 min at room temperature in microtiter plates. The polymerization reaction was initiated by the addition of template:primer substrate (final concentration, 16.6 nM), and dNTPs (final concentrations were 2 μM for deoxycytidine triphosphate [dCTP], deoxyguanosine triphosphate [dGTP], and dATP and 66.6 nM for ruthenylated deoxyuridine triphosphate [Ru-dUTP]). After 90 min of incubation at 37 °C, reactions were quenched by the addition of ethylenediaminetetraacetic acid (EDTA, 25 mM). The resulting mixture was incubated for an additional 5 min at room temperature, followed by transfer of the solution (50 μL) to a blocked avidin plate from Meso Scale Discovery (Meso Scale Diagnostics; Rockville, MD, USA). The mixtures were incubated at room temperature for 60 min prior to the quantification of the reaction product via an ECL 6000 imager instrument. All IC_50_ values were relative as they were highly dependent on assay conditions.

### Evaluation of antiviral activity in a single-cycle assay in PBMCs

To evaluate the ability of inhibitors to prevent HIV-1 infection in primary cells, human PBMCs obtained from Biological Specialty Corporation (Colmar, PA, USA) were used to assess antiviral activity. The proviral variants used for these studies were generated by site-directed mutagenesis in a proviral vector derived from NL43 [52] that encodes GFP (pNLG1-P2A-GFP). Virus was produced by transfection in 293T cells.

PBMCs were activated with phytohemagglutinin (PHA) at 5 μg/mL in Roswell Park Memorial Institute (RPMI) 1640 medium containing 10% heat-inactivated fetal bovine serum (FBS) for 2–3 days. Compound plates were prepared by dispensing compounds dissolved in DMSO into 384-well poly-d-lysine-coated plates (0.2 μL per well) using an Echo acoustic dispenser (Labcyte; San Jose, CA, USA). Each compound was tested in a 10-point serial 3-fold dilution (typical final concentrations: 5,000 to 0.1 nM). Controls included no inhibitor (DMSO only) and a combination of 3 antiviral agents, efavirenz, indinavir, and the integrase strand transfer inhibitor, L-002254051, at final concentrations of 4 μM each. PHA-activated human PBMCs were washed and resuspended in RPMI 1640 containing 10% NHS and 30 units per mL of interleukin-2 (IL-2) at 6 × 10^5^ cells per mL. PBMCs were added to the compound plates and cultured for 24 h at 37 °C, 5% CO_2_, and 90% humidity. The appropriate HIV-1 variant was added to the PBMCs and cultured at 37 °C, 5% CO_2_, and 90% humidity for an additional 24 h.

HIV-1–infected cells were quantified by counting the number of GFP cells in each well using an Acumen eX3 scanner (SPT Labtech; Melbourn, UK). IC_50_ values were determined by nonlinear 4-parameter curve fitting of the data.

### Assessing the persistence of MK-8527 antiviral activity in MT4-GFP cells and PBMCs

MT4-GFP cells or 72-h PHA-stimulated human PBMCs were cultured and plated onto 384-well poly-d-lysine coated plates containing 10-point serial 3-fold compound dilutions (in DMSO). Assay media for MT4-GFP cells contained RPMI 1640, GlutaMAX (Thermo Fisher Scientific; Waltham, MA, USA), 10% heat-inactivated NHS and 1% penicillin-streptomycin, whereas 20 IU/mL IL-2 was added to the assay media for PBMC experiments. Cells were incubated with compounds at 37 °C, 5% CO_2_, and 90% humidity for 24 h to allow for nucleoside uptake and phosphorylation. At 24 h after cell plating, the plates were centrifuged at 300*g* for 5 min to ensure maximum attachment of cells to the poly-d-lysine coating. Assay media in each well were then evacuated and the cells resuspended using the gentle setting on the BlueWasher (BlueCatBio; Lebanon, NH, USA). Centrifugation and wash steps were repeated to ensure removal of extracellular compound.

Following compound washout, cells were either infected immediately (Fig 3; variable A) or at 48 h post-washout (Fig 3; variable B). MT4-GFP cells were infected with WT R8 virus, and PBMCs were infected with pNLG1-P2A-GFP. The number of GFP cells in each well were quantified at 24 h post-infection using an Acumen eX3 scanner (SPT Labtech; Melbourn, UK). Virus input for each cell type was determined by targeting approximately 300 and 600 GFP-positive cells per well at 24 h after infection for control wells in MT4-GFP and PBMCs, respectively. IC_50_ values were determined by nonlinear 4-parameter curve fitting of the data. A persistence ratio was calculated for each compound by dividing the IC_50_ from variable B (IC_50_B) by the IC_50_ from variable A (IC_50_A).

### Evaluation of the antiviral activity against HIV-1 and HIV-2 in CEM-SS cells

The antiviral activity of MK-8527 against HIV-1 and HIV-2 was determined in a cytoprotection assay in CEM-SS cells at ImQuest BioSciences (Fredrick, MD, USA). Briefly, CEM-SS cells (National Institute of Health AIDS Research and Reference Reagent Program) were passaged in RPMI 1640 supplemented with 10% heat-inactivated FBS, 2 mM l-glutamine, 100 U/mL penicillin and 100 µg/mL streptomycin prior to use in the antiviral assay. On the day preceding the assay, the cells were split 1:2 to ensure they were in an exponential growth phase at the time of infection. The cells were resuspended at 2.5 × 10^3^ cells per well in cell culture medium and added to the drug-containing microtiter plates at 50 µL per well. MK-8527 was tested in triplicate for each experiment, with 12 test concentrations added at 100 µL per well.

Lymphocyte-tropic virus strains HIV-1_IIIB_ or HIV-2_ROD_ (NIH AIDS Research and Reference Reagent Program) were diluted into tissue culture medium such that the amount of virus added to each well in a volume of 50 mL corresponded to the amount determined to yield 85% to 95% cell killing in the untreated virus control wells at 6–7 days. Virus was added immediately after cells were added to the 96-well microtiter plates. Following incubation at 37 °C in a 5% CO_2_ incubator for 6 days, the test plates were stained with the tetrazolium dye XTT (2,3-bis(2-methoxy-4-nitro-5-sulfophenyl)-5-[(phenylamino)carbonyl]-2H-tetrazolium hydroxide) and quantified spectrophotometrically at 450/650 nm with a *V*_max_ plate reader (Molecular Devices; San Jose, CA, USA).

The IC_50_ for MK-8527 antiviral activity was determined using 4-parameter curve fit calculations.

### Antiviral activity of MK-8527 against HIV-1 subtypes

Compounds were evaluated for antiviral activity against a panel of clinical isolates representing multiple HIV-1 subtypes at Monogram Biosciences (South San Francisco, CA, USA). The isolates were selected based on an absence of resistance-associated mutations in RT and each was constructed as a pooled resistance test vector designed to preserve the quasispecies present in the initial clinical isolate. Fold change in concentration of test compound required to inhibit cytopathic effect by 50% (EC_50_) against vectors containing the WT protease and RT sequences derived from various HIV-1 subtypes in human plasma versus a drug-sensitive reference virus (strain CNDO) containing the protease and RT from the NL4-3 strain of HIV-1 in the PhenoSense assay was determined [40].

### Antiviral activity of MK-8527 against a diverse panel of viruses

MK-8527 activity against a diverse panel of viruses was assessed at Utah State University (Logan, UT, USA) based on the ability of the compound to prevent virus from causing viral cytopathic effect in cell culture. Briefly, compounds (MK-8527 or a control compound specific for each virus) were tested for in vitro antiviral activity against adenovirus-5 in A549 cells; Tacaribe virus in Vero cells; Rift Valley fever virus, West Nile virus, herpes simplex virus-1, herpes simplex virus-2, poliovirus-1, and Venezuelan encephalitis virus in Vero 76 cells; murine norovirus in RAW cells; dengue virus-2 in Huh7 cells; influenza A/California/07/2009(H1N1) and influenza B/Brisbane/60/2008 in MDCK cells; and respiratory syncytial virus-A2 and enterovirus-68 in RD cells. Cytopathic effect was monitored 3–7 days after infection and treatment, with a neutral red dye with optical density read on a spectrophotometer at 540 nm. Optical densities were converted to percentage of cell controls and normalized to the virus control, then the EC_50_ was calculated by regression analysis. The concentration of compound that would cause 50% cell death (CC_50_) in the absence of virus was similarly calculated.

### Uptake and phosphorylation assay to measure the concentration of MK-8527-TP in PBMCs

To determine the amount of MK-8527 phosphorylated forms produced after treatment of PBMCs, uptake and phosphorylation assays were performed. PBMCs were activated with 5 µg/mL PHA in RPMI 1640, 10% FBS, 100 units/mL penicillin, and 100 µg/mL streptomycin at 1.2 × 10^6^ cells/mL. All incubations were in a humidified incubator at 37 °C and with 5% CO_2_. After 3–4 days in culture, media containing PHA were removed by centrifugation at 300*g* for 10 min. Cells were resuspended at 2 × 10^6^ cells/mL in RPMI 1,640, 10% NHS, 100 units/mL penicillin, 100 µg/mL streptomycin, and 20 units/mL IL-2. A total of 7.5 × 10^6^ to 2 × 10^7^ cells were transferred to a miniature bioreactor tube. All experiments were performed in triplicate, with MK-8527 added at a final concentration of 0.21 nM or 2.1 nM and a final DMSO concentration of 0.3% (v/v percentage) and incubated for 24–72 h. At the appropriate time point, the miniature bioreactor tubes were centrifuged at 300*g* for 7 min at 4 °C, and the pellet was washed in wash buffer (40 mL ice-cold phosphate buffered saline [PBS] with 2% FBS) and centrifuged again. Cell pellets were gently resuspended with 10 mL of wash buffer, and 1 mL of the cell suspension was removed for cell count. The remaining cell suspension was centrifuged again, and cell pellets were resuspended in 1 mL wash buffer and transferred to a microcentrifuge. Cells were centrifuged at 6,797*g* for 5 min at 4 °C. Pellets were resuspended in 100 µL cold 70% methanol/30% distilled water by pipetting up and down, and samples were frozen on dry ice and stored at −80 °C until analysis by LC–MS/MS.

### Bioanalytical process for measurement of MK-8527-TP levels in PBMCs treated with MK-8527 in vitro or isolated from rhesus monkeys dosed with MK-8527

The intracellular concentration of MK-8527-TP was determined by an LC–MS/MS assay. LC–MS/MS analysis was performed on a Acquity UPLC system (Waters Corporation; Milford, MA, USA) interfaced to an API-5500 mass spectrometer using a TurboIonSpray source (Sciex, Framingham, MA, USA) in positive ionization mode. Separation of MK-8527-TP was achieved on a BioBasic AX column (2.1 × 100 mm, 5 µm; Thermo Fisher Scientific) at 45 °C using gradient elution with mobile phases consisting of 70% water to 30% acetonitrile containing 10 mM ammonium acetate (solvent A) and 70% water to 30% acetonitrile containing 20 mM ammonium acetate, 1.5% ammonium hydroxide (NH_4_OH) (v/v), and 10% isopropyl alcohol (IPA) (v/v) (solvent B) at a flow rate of 0.75 mL/min. The chromatography was run as follows: The column was equilibrated with 97% A, and after sample injection 97% A was maintained for 0.10 min before concentration was increased linearly to 100% B until 3.00 min, followed by a hold at 100% B until 3.50 min. The column was then washed by alternating linear ramps from 97% A to 100% B up to 4.10 min, then returned to the initial conditions at 4.30 min and kept for an additional 1.20 min. The total run time was 5.50 min. Quantification was performed by monitoring the transitions of *m*/*z* 548.9 to 169.1 for MK-8527-TP. The method was linear across a concentration range of 1–5,000 nM in cell samples across species. The method’s lower limit of quantification was between 1 and 5 nM depending on the assay volumes of cell suspension. Analyte concentrations in cell samples across species were determined from the standard curves using linear regression with 1/×2 weighting.

Concentrations of intracellular phosphorylated forms were expressed in picomoles per 10^6^ cells by using the molar concentration of each compound as determined by LC–MS/MS and the number of viable cells included in each 100-µL sample. Linear regression analysis to extrapolate the concentration of MK-8527-TP at the 0.21 nM IC_50_ was performed in GraphPad Prism 8.1.1 (GraphPad; San Diego, CA, USA).

### Human DNA polymerase inhibition assays

The activities of human DNA polymerases α, β, and γ were measured in radiolabeled nucleotide incorporation assays. TP was prepared as a 10-point titration with 3-fold serial dilution in water. Reaction buffers for the DNA polymerase α and β assays both contained 20 mM Tris pH 7.5, 10 mM MgCl_2_, 50 μM EDTA, 200 μg/mL bovine serum albumin (BSA), 2 mM dithiothreitol (DTT), 1.6 μM each of dNTP (dATP, dCTP, dGTP, and dTTP), 20 μCi/mL [α33P]dATP, 0.1 mg/mL gapped activated fish sperm DNA template, test compound, and 0.005 U/μL DNA polymerase α or 0.0025 U/μL DNA polymerase β. The reaction buffer for DNA polymerase γ contained 20 mM Tris pH 8, 10 mM MgCl_2_, 50 mM KCl, 100 μg/mL BSA, 2 mM β-mercaptoethanol, 10 μM of each dNTP (dATP, dCTP, dGTP, and dTTP), 20 μCi/mL [α33P]dATP, 0.04 mg/mL gapped activated fish sperm DNA template, test compound, and 0.0003 μg/μL DNA polymerase γ. The 50-μL reactions were incubated at room temperature for 30 min (for β and γ) or 90 min (for α) before terminating with the addition of 50 μL 500 mM EDTA. The reaction mixtures were transferred to a Millipore DE81 filter plate (EMD Millipore; Hayward, CA), washed with 0.5 M sodium phosphate pH 7.0, and dried. After addition of 40 μL scintillant to each well, the incorporation of labeled dATP was determined with a microplate counter (TopCount NXT HTS; PerkinElmer, Shelton, CA, USA). Determinations of the activity at each inhibitor concentration (top concentration, 200 μM) were used to calculate compound IC_50_ values, which were determined by nonlinear 4-parameter curve fitting.

### Off-target assays to evaluate specificity

MK-8527 and MK-8527-TP were evaluated at 10 µM final concentration at Eurofins Panlabs (Taipei, Taiwan) for activity against a panel of 114 enzyme, radioligand binding, and cellular assays, including 70 G-protein coupled receptor, 13 ion channel, 7 kinase, 7 nuclear hormone receptor, 6 transporter, 4 protease, 2 neurotransmitter metabolism, 2 lipid metabolism, 2 oxidoreductase, and 1 phosphodiesterase assays.

### Assessment of the translocation state of RT by iron footprinting Assays

The translocation state of RT was assessed with a site-specific iron footprinting assay adapted from previous work [23,24]. Primer and template used for this assay are described in Fig 7A. The presence of Fe^2+^ ions in the reaction generated hydroxyl radicals that cleaved the DNA template in a site-specific manner at the nucleotide closest to the RNase H active site. In addition to an RT inhibitor in the reaction, dTTP, the subsequent dNTP, was provided in dose titration to force the translocation of the 3′-primer terminus from the pre-translocation N-site to the post-translocation P-site.

The reaction was conducted as a sequential addition of the following reagents to the 96-well polymerase chain reaction plate: HIV RT (final concentration, 1,200 nM), either MK-8527-TP (final concentration, 1 µM), ISL-TP (final concentration, 1 µM), or ddATP (final concentration, 5 µM), Td43/Pd30 pre-annealed primer and template (final concentration, 200 nM based on Cy3-labeled Td43 concentration), and dTTP titrated at various concentrations. All reagent stocks were in a base assay buffer consisting of 120 mM Tris pH 7.5, 20 mM NaCl, and 6 mM MgCl_2_ in nuclease-free water. The reactions were incubated at room temperature for 7 min before the addition of ammonium iron sulfate (final concentration, 0.125 mM) and DTT (final concentration, 1 mM) in nuclease-free water. The reaction was then heated to 37 °C for 5 min before quenching 1:1 with gel-loading dye followed by heating at 95 °C for 10 min. Samples were stored at −20 °C until being resolved by polyacrylamide gel electrophoresis with 7 M urea.

### Assessment of immediate or delayed chain termination by primer extension assay

A fluorescently labeled primer extension assay with RT was done to monitor chain termination by MK-8527-TP. The reaction protocol was adapted from previous work [23,24]. Primers and template used for this assay are described in Fig 8A.

The reaction was a sequential addition of the following reagents to the 96-well polymerase chain reaction plate: 2.5× HIV RT (final concentration, 100 nM), 5× Pd18+5/Td26 pre-annealed primer and template (final concentration, 200 nM based on the Cy3-labeled Pd18+5 concentration), and 5× dNTP with and without various concentrations of test compound (1 µM final concentration of each of the dNTPs with MK-8527-TP, ISL-TP or ddATP dilutions prepared in the 5× dNTP buffer stock to maintain consistent dNTP concentrations across reactions). All reagent stocks were in a base assay buffer consisting of 50 mM Tris pH 7.5 and 50 mM NaCl in nuclease-free water. The RT primer extension assay was initiated with the addition of 5× MgCl_2_ (30 mM MgCl_2_ diluted in 50 mM Tris pH 7.5 and 50 mM NaCl in nuclease-free water) and then proceeded for 15 min at 37 °C before being quenched with the addition of 2× gel loading dye. Samples were then heated to 95 °C for 10 min prior to storage at −20 °C until being resolved by polyacrylamide gel electrophoresis with 7 M urea.

### PK in rats and rhesus monkeys

All animal procedures were conducted according to the highest ethical standards using procedures approved by the Institutional Animal Care and Use Committee of Merck & Co., Rahway, NJ, USA (APS 2016-600774-FEB). Fasted male Wistar Hannover rats (*n* = 3 in each arm) weighing approximately 325 g were used for the PK studies. All blood samples were collected in EDTA-coated tubes at the time points specified below. For the IV arm of the study, a cannula was implanted in the jugular vein for dose administration and blood sampling. MK-8527 was administered as a bolus at 1 mg/kg in DMSO and saline at a 50:50 ratio. Blood samples were collected serially at 0, 0.03, 0.13, 0.25, 0.5, 1, 2, 4, 6, 8, and 24 h following dose administration. For the oral arm of the study, a solution of MK-8527 was administered by oral gavage at 2 mg/kg in 0.5% methylcellulose in water to fasted rats. Blood samples were collected serially at 0.25, 0.5, 1, 2, 4, 7, 12, 18, 24, 30, and 48 h following dose administration.

Fasted rhesus monkeys (*n* = 3 in each arm) weighing 5.3–9.1 kg were used for the MK-8527 PK studies. For the IV arm of the study, MK-8527 was administered as a bolus via the cephalic vein at 0.5 mg/kg in 30% Captisol (Ligand Pharmaceuticals, Lawrence, KS, USA). Blood samples were collected serially from the femoral vein in EDTA-coated tubes at 0.03, 0.13, 0.25, 0.5, 1, 2, 4, 6, 24, 30, 48, and 72 h. For the oral arm of the study, a solution of MK-8527 was administered by oral gavage at 1 mg/kg in 0.5% methylcellulose to fasted monkeys. The blood samples were collected serially at 0.25, 0.5, 1, 2, 4, 6, 24, 30, 48, and 72 h following dose administration. Monkeys were dosed, bled, and monitored in study chairs for up to 6 h post-dose. After the 6-h time point, they were returned to their cages and placed in a bleed chair for the subsequent time points. No anesthesia was used. As enrichment, animals were pair housed with conspecifics in cages with perches. They received toys which were rotated every two weeks, and they had access to TV for a minimum of 3 h per week. Animals were fed certified primate diet (LabDiet 5048) and given a vitamin C supplement daily. In addition, animals received rotational fruit and vegetables as well as a foraging mix daily. All blood samples were centrifuged at 2,000*g* for 10 min, and the supernatant plasma samples were removed and stored at −70 °C until further analysis.

### Determination of MK-8527 concentration in plasma

The concentrations of MK-8527 in plasma were determined by LC–MS/MS assays following a protein precipitation step. Aliquots (50 µL) of plasma were precipitated by addition of 150 µL of acetonitrile solution-containing internal standard (IS), followed by centrifugation at 3,000 rpm for 10 min. A 75-µL aliquot of the supernatant was transferred into a 96-well plate, and 5 µL of each sample was used for analysis. LC–MS/MS analysis was performed on a Transcend LC system with HTS PAL CTC autosampler (Thermo Fisher Scientific, Waltham, MA, USA) interfaced to an API-5000 mass spectrometer using the turbo ion spray interface (Sciex, Framingham, MA, USA). Separation of MK-8527 was achieved on an Xselect HSS XP T3 column (50 × 2.1 mm, 2.5 µm; Waters) using a mobile phase consisting of 0.1% formic acid in water (solvent A) and 0.1% formic acid in acetonitrile (solvent B) at a flow rate of 0.75 mL/min.

The chromatography for plasma samples was run using gradient elution as follows: the column was equilibrated with 100% A. After sample injection, solvent A was maintained at 95% for 0.25 min before solvent B was increased linearly to 95% B over 1.5 min. Solvent B was maintained at 95% B for 0.92 min before the fraction of solvent B was returned to the initial conditions and kept for an additional 1.5 min. The total run time was 4.15 min. Quantification was done by monitoring the transition of *m*/*z* 309 to *m*/*z* 169 or 133 for MK-8527. The method was linear across a concentration range of 2–10,000 ng/mL in plasma.

### Isolation of PBMCs from blood for PK analysis

PBMCs were isolated from heparinized monkey blood using the following protocol. To begin, blood samples (approximately 12–18 mL) were diluted with an equal volume of calcium- and magnesium-free PBS at pH 7.4 and gently mixed. Separation of PBMCs from the diluted blood was achieved using 50 mL AccuSpin tubes loaded with Histopaque-1077 lymphocyte separation medium (both: Thermo Fisher Scientific). Samples were centrifuged at 800*g* for 15 min at room temperature. The resulting plasma/buffer supernatant was removed without disturbing the PBMC interface. Next, the PBMC interface was removed with a pipette, and the cells were washed by the addition of calcium- and magnesium-free PBS (pH 7.4) to a final volume of 15 mL. PBMCs were harvested by centrifugation at 250*g* for 20 min at room temperature. The supernatant was decanted with care taken not to disturb the PBMC pellet, and the wash step was repeated once (250* × g* for 15 min). After the second wash, the cell pellet was resuspended in 7 mL of calcium- and magnesium-free PBS, and an aliquot was removed for cell counting. PBMCs were harvested from the remaining cell suspension by centrifugation at 250*g* for 15 min at room temperature. The supernatant was removed, and the cell pellet was resuspended in 0.1–0.3 mL of methanol and water at a 70:30 ratio. Cell suspensions were stored at 70 °C prior to analysis.

### Determination of the crystal structure of RT in complex of MK-8527 and primer:template

Protein expression, purification, and crystallization: The HIV-RT p51 (C280S)/p66 (Q258C/C280S) complex containing a thioalkylated primer/template DNA duplex was prepared as previously described [24,26]. The oligonucleotides (primer: 5′-ACA GTC CCT GTT CGG G*CG CC-3′ where G* has a thioalkyl tether, and template: 5′-ATG GTC GGC GCC CGA ACA GGG ACT GTG-3′) were chemically synthesized by TriLink BioTechnologies (San Diego, CA, USA). This complex was also incubated with 2′,3′-dideoxyguanosine 5′-triphosphate to terminate the primer strand and allow capture of the MK-8527-TP pre-catalytic complex, analogous to the earlier procedure described by Salie and colleagues to capture the pre-catalytic complex with 4′-ethynyl-2-fluoro-2′-deoxyadenosine (islatravir) [26]. The HIV-RT/DNA complex was eluted from size exchange chromatography into buffer containing 25 mM Tris pH 8.0, 100 mM NaCl and concentrated to 22 mg/mL. Apo crystals were grown in screw-cap plates at 18 °C by hanging drop vapor diffusion over a reservoir solution containing 8% PEG 4000, 25 mM MES pH 7.0, and 5 mM MgSO_4_ at a 2:1 protein:precipitant ratio. A cluster of apo crystals was transferred to a fresh drop of mother liquor supplemented with 4 mM MK-8527-TP and soaked for 5 days. Following soaking, the crystals were dehydrated over a period of 1 week by equilibrating the sample over a series of reservoirs with increasing concentration of PEG 4000 (12% to 35%). The final crystal was harvested from 35% PEG 4000, 25 mM MES pH 7.0, 5 mM MgSO_4_ and flash frozen in liquid nitrogen for data collection.

### Structure determination

Diffraction data were collected at the Advanced Photon Source on the Industrial Macromolecular Crystallography Association – Collaborative Access Team beamline (17-ID) and subsequently processed using autoPROC and STARANISO due to anisotropic diffraction [53]. Initial refinement was conducted using the apo-form of PDB:5J2M as a starting model. Model building and refinement were conducted in Coot [54] and Phenix [55]. A summary of the diffraction and refinement statistics can be found in S1 Table.

## Supporting information

S1 Text Supporting materials and methods. (DOCX)

S1 Table Data collection and refinement statistics. Statistics for the highest resolution shell are shown in parentheses.
(DOCX)

S1 Raw Images Original uncropped blots for Fig 7B–7D. **Iron footprinting assay to evaluate the position of RT on the primer and template.** ddATP, dideoxyadenosine triphosphate; dTTP, deoxythymidine triphosphate; ISL, islatravir; RT, reverse transcriptase; TP, triphosphate.
(PDF)

S2 Raw Images Original uncropped blots for Fig 8B–8D. **HIV-RT primer extension assay to evaluate the chain termination mechanism of MK-8527-TP compared with ISL-TP and ddATP (an NRTI).** ddATP, dideoxyadenosine triphosphate; dTTP, deoxythymidine triphosphate; ISL, islatravir; NRTI, nucleos(t)ide reverse transcriptase inhibitor; RT, reverse transcriptase; TP, triphosphate.
(PDF)

S1 Data Source Data for Fig 6. Activity of MK-8527 against HIV-1 subtypes. Data are presented as fold change in IC_50_ for each clinical isolate compared to the internal CNDO control virus. IC_50_, half maximal inhibitory concentration; WT, wild-type.
(XLSX)

S2 Data Source Data for Fig 10. Concentration-vs-time profiles of MK-8527 in plasma (A) and MK-8527-TP in PBMCs (B) following oral administration to rhesus monkeys at 50 mg/kg. PBMCs, human peripheral blood mononuclear cells; TP, triphosphate.
(XLSX)

## Abbreviations

ARV: antiretroviral
CH_2_: methylene
CLP: plasma clearance
CTG: CellTiter-Glo
DCT: delayed chain termination
DMSO: dimethyl sulfoxide
ddATP: dideoxyadenosine triphosphate
dNTP: deoxynucleotide triphosphate
dTTP: deoxythymidine triphosphate
EC50: concentration of test compound required to inhibit cytopathic effect by 50%
*F*: oral bioavailability
FBS: fetal bovine serum
GFP: green fluorescent protein
hpi: hours post-infection
IBX: 2-iodoxybenzoic acid
I_C_50: half maximal inhibitory concentration
ICT: immediate chain termination
ISL: islatravir
ISL-TP: islatravir triphosphate
IV: intravenous
LC–MS/MS: liquid chromatography with tandem mass spectrometry
Me: methyl
MeCN: acetonitrile
MeOH: methanol
NC: not calculated
NHS: normal human serum
NRTI: nucleos(t)ide reverse transcriptase inhibitor
NNRTI: non-nucleoside reverse transcriptase inhibitor
NRTTI: nucleoside reverse transcriptase translocation inhibitor
PBMC: peripheral blood mononuclear cell; PBS, phosphate buffered saline
PK: pharmacokinetics
RT: reverse transcriptase
SAR: structure–activity relationship
SD: standard deviation
*t*_½_: half-life
TBAF: tetrabutylammonium fluoride
TBSCl: tert-butyldimethylsilyl chloride
THF: tetrahydrofuran
TFA: trifluoroacetic acid
OTBS: O-tert-butyldimethylsilyl
TP: triphosphate
*V*dss: steady-state volume of distribution
WT: wild-type
