# Supplementary material for: MK-8527 is a novel inhibitor of HIV-1 reverse transcriptase translocation with potential for extended-duration dosing
Source: PLoS Biol. 2025 Aug 26;23(8):e3003308. doi: 10.1371/journal.pbio.3003308 (PMC12380353; doi:10.1371/journal.pbio.3003308)
Supplement: S1 Text — (DOCX) [file pbio.3003308.s001.docx]

**Supporting Information:**

**Supporting Materials and Methods**

**Discovery of MK-8527, a novel potent inhibitor of HIV-1 reverse transcriptase translocation with potential for extended-duration dosing**

**Table of Contents**

Abbreviations **S2**

General Techniques and Reagents for Compound Synthesis **S4**

Synthesis of **MK-8527 (Compound 12)** **S6**

Compound Synthesis Examples **S15**

**Abbreviations and Acronyms**

| AcOH = acetic acid | MES = 2-(*N*-morpholino)ethanesulfonic acid |
| --- | --- |
| Ar = argon | m = multiplet |
| BSA = benzene sulfonic acid | MHz = megahertz |
| br = broad | mL = milliliter |
| d = doublet | mol = moles |
| dd = doublet of doublets | mmol = millimoles |
| DMAP = dimethylamino pyridine | NaH = sodium hydride |
| DMF = *N*,*N*-dimethylformamide | NHS = normal human serum |
| DMSO = dimethyl sulfoxide | nm = nanometer |
| DNA = deoxyribonucleic acid | NMR = nuclear magnetic resonance |
| EtOAc = ethyl acetate | PBS = phosphate buffered saline |
| EtOH = ethanol | ppm = parts per million |
| HCl_aq_ = aqueous hydrochloric acid | PEG = polyethylene glycol |
| HCl_g_ = hydrogen chloride gas | rac = racemic |
| HIV = human immunodeficiency virus | RT = reverse transcriptase |
| HPLC = high performance liquid chromatography | s = singlet |
| Hz = hertz | SiO_2_ = silicon dioxide |
| IBX = 2-iodoxybenzoic acid | t = triplet |
| *i*PrOH = isopropanol | TBAF = tetrabutylammonium fluoride |
| LC-MS = liquid chromatography-mass spectrometry | TBSCl = t-butyldimethylsilyl chloride |
| LRMS = low resolution mass spectroscopy | THF = tetrahydrofuran |
| L = liter | TFA = trifluoroacetic acid |
| LDA = lithium diisopropylamide | TLC = thin layer chromatography |
| Me = methyl | TP = triphosphate |
| MeCN = acetonitrile | TMS = tetramethylsilane |
| MeOH = methanol | UV = ultraviolet |

**General Techniques and Reagents for Compound Synthesis**

Reactions sensitive to moisture or air were performed under nitrogen or argon (Ar) using anhydrous solvents and reagents. The progress of reactions was determined by either analytical thin layer chromatography (TLC) usually performed with pre-coated TLC plates (MilliporeSigma), silica gel 60F-254, layer thickness 0.25 mm or liquid chromatography-mass spectrometry (LC-MS). Typically, the analytical LC-MS system used consisted of a Waters ZQ platform with electrospray ionization in positive ion detection mode with an Agilent 1100 series HPLC with autosampler. The column typically used was a Water Xterra MS C18, 3.0×50 mm, 5 μm. The flow rate was 1 mL/min with an injection volume of 10 μL. Ultraviolet (UV) detection was in the range 210–400 nm. The mobile phase generally consisted of solvent A (water plus 0.06% trifluoroacetic acid [TFA]) and solvent B (acetonitrile [MeCN] plus 0.05% TFA) with a gradient of 100% solvent A for 0.7 minutes changing to 100% solvent B over 3.75 minutes, which was maintained for 1.1 minutes and then reverted to 100% solvent A over 0.2 minutes.

Preparative high-performance liquid chromatography (HPLC) purifications were usually performed using a UV and/or mass spectrometry (MS) directed system. Typically, these were performed on a Waters Chromatography Workstation configured with LC-MS system consisting of: Waters ZQ single quad MS system with Electrospray Ionization, Waters 2525 Gradient Pump, Waters 2767 Injector/Collector, Waters 996 PDA Detector, the MS Conditions of: 150–750 amu, Positive Electrospray, Collection Triggered by MS, and a Waters Sunfire C-18 5 micron, 30 mm (id) x 100 mm column; however, other systems were also employed. The mobile phases consisted of mixtures of MeCN (10–100%) in water containing a basic or acidic modifier. Concentration of solutions was carried out on a rotary evaporator under reduced pressure. Flash chromatography was usually performed using a Biotage Flash Chromatography apparatus on silica gel (32–63 μM, 60 Å pore size) in pre-packed cartridges of the size noted. ^1^H NMR spectra were acquired at 300–600 megahertz (MHz) spectrometers in deuterated solvents as noted. Chemical shifts were reported in parts per million (ppm). Tetramethylsilane (TMS) was used as internal reference in deuterated chloroform (CD_3_Cl) solutions, and residual methanol (MeOH) peak or TMS was used as internal reference in *d6*-dimethyl sulfoxide (DMSO) solutions. Coupling constants (*J*) were reported in hertz (Hz).

Several methods for preparing the compounds mentioned in this manuscript are described in the examples below. Starting materials and intermediates were purchased commercially from common catalog sources or were made using known procedures, or as otherwise described. In some cases, the order of which the steps of the reaction schemes were carried out varied to facilitate the reaction or to avoid unwanted reaction products.

**Synthesis of MK-8527 (Compound 12)**

MK-8527 (*(2R,3S,5R)-5-(4-amino-2-chloro-7H-pyrrolo[2,3-d]pyrimidin-7-yl)-2-ethynyl-2-(hydroxymethyl)tetrahydrofuran-3-ol)_;_* synthesis is also summarized in Fig 9 in the main manuscript)

**Fig A. Synthesis of MK-8527.^a^**

^a^Reagents and conditions: (a) acetyl chloride, methanol (MeOH); (b) *p-*toluoyl chloride, pyridine; (c) HCl, diethyl ether; (d) 2,6-dichloro-7-deazapurine, sodium hydride (NaH), acetonitrile (MeCN); (e) ammonia (NH_3_) in isopropanol, sodium methoxide (NaOMe) in MeOH; (f) *tert*-butyldimethylsilyl chloride (TBSCl), imidazole, *N,N-*dimethylformamide (DMF); (g) trifluoroacetic acid (TFA), tetrahydrofuran (THF), H_2_O; (h) 2-iodoxybenzoic acid (IBX), MeCN, dimethyl sulfoxide (DMSO); (i) 1. formaldehyde, NaOH, 2. NaBH_4_, ethanol (EtOH); (j) IBX, MeCN; (k) Bestmann−Ohira Reagent, K_2_CO_3_; (l) tetrabutylammonium fluoride (TBAF), THF.

*Step 1: Synthesis of (2R,3S)-2-(hydroxymethyl)-5-methoxytetrahydrofuran-3-ol* **(Compound 22)**

To a stirred solution of (4*S*,5*R*)-5-(hydroxymethyl)tetrahydrofuran-2,4-diol (7.0 g, 52.2 mmol) in anhydrous MeOH (105 mL) under Ar atmosphere, acetyl chloride (0.614 g, 7.83 mmol) was added dropwise and stirred at 0 ºC over 5 minutes. The resulting mixture was stirred at 0 ºC for a further 3 hours. Next, solid NaHCO_3_ was added to neutralize the reaction mixture. The organic phase was collected by filtration and then concentrated under reduced pressure. The resulting residue was purified by chromatography on SiO_2_ (isocratic elution with 5% MeOH/CH_2_Cl_2_) to provide the title compound (6.5 g, 43.9 mmol, 84% yield) as a mixture of isomers. ^1^H NMR: (300 MHz, CDCl_3_, ppm): δ 4.98–5.04 (m, 1H), 4.21–4.23 (m, 0.4H), 4.07–4.09 (m, 0.6H), 3.83–3.89 (m, 1H), 3.50–3.66 (m, 2H), 3.27–3.32 (m, 3H), 2.23–2.29 (m, 0.6H), 2.08–2.12 (m, 0.4H), 1.98–2.03 (m, 0.4H), 1.77–1.83 (m, 0.6H).

*Step 2: Synthesis of (2R,3S)-5-methoxy-2-(((4-methylbenzoyl)oxy)methyl)tetrahydrofuran-3-yl 4-methylbenzoate* **(Compound 23)**

To a stirred solution of Compound 22 (6.5 g, 43.9 mmol) in dry pyridine (45 mL) under Ar atmosphere, *p-*toluoyl chloride (20.35 g, 132 mmol) was added dropwise at 0 ºC over 15 minutes. The resulting mixture was gradually heated to 30 ºC and then stirred for 16 hours. The resulting solution was concentrated under reduced pressure and the residue was diluted with ethyl acetate (EtOAc) (400 mL), and then washed with HCl_aq_ (1 M, 2x 100 mL), saturated aqueous NaHCO_3_ (2x 100 mL), and brine (100 mL). The organic phase was collected, dried over anhydrous Na_2_SO_4_, filtered, and concentrated under reduced pressure. The resulting residue was purified by chromatography on SiO_2_ (isocratic elution with 5% EtOAc/petroleum ether) to provide the title compound (13.0 g, 33.8 mmol, 77% yield) as a mixture of isomers. ^1^H NMR: (300 MHz, CDCl_3_, ppm): δ 7.90–7.99 (m, 4H), 7.20–7.26 (m, 4H), 5.58–5.61 (m, 0.47H), 5.39–5.42 (m, 0.55H), 5.18–5.24 (m, 1H), 4.46–4.65 (m, 3H), 3.43 (s, 1.61H), 3.36 (s, 1.33H), 2.52–2.60 (m, 1H), 2.17–2.43 (m, 7H).

*Step 3: Synthesis of (2R,3S,5R)-5-chloro-2-(((4-methylbenzoyl)oxy)methyl)tetrahydrofuran-3-yl 4-methylbenzoate* **(Compound 24)**

HCl_g_ was introduced to a stirred solution of Compound 23 (4.0 g, 10.41 mmol) in anhydrous diethyl ether (20 mL) in a 100 mL three-necked round-bottom flask at 0 ºC. The product gradually precipitated out over period of 40 minutes. The solid precipitate was filtered and washed with anhydrous diethyl ether (3x 20 mL) and dried under high vacuum for 2 hours to provide the title compound (2.7 g, 6.94 mmol, 66.7% yield) as a mixture of isomers. ^1^H NMR: (300 MHz, CDCl_3_, ppm): δ 7.96 (d, *J*=9.0 Hz, 2H), 7.90 (d, *J*=8.1 Hz, 2H), 7.19–7.27 (m, 4H), 6.47 (d, *J*=5.1 Hz, 1H), 5.54–5.58 (m, 1H), 4.85 (dd, *J*=3.3, 7.2Hz, 1H), 4.68 (dd, *J*=3.3, 12.0 Hz, 1H), 4.59 (dd, *J*=4.2, 12.0 Hz, 1H), 2.83–2.92 (m, 1H), 2.74 (d, *J*=15.0 Hz, 1H), 2.42 (d, *J*=3.6 Hz, 6H).

*Step 4: Synthesis of (2R,3S,5R)-5-(2,4-dichloro-7H-pyrrolo[2,3-d]pyrimidin-7-yl)-2-(((4-methylbenzoyl)-oxy)methyl)tetrahydrofuran-3-yl 4-methylbenzoate* **(Compound 25)**

To a stirred suspension of NaH (60% dispersion in mineral oil, 0.509 g, 12.73 mmol) in anhydrous MeCN (125 mL), 2,4-dichloro-7*H*-pyrrolo[2,3-d]pyrimidine (2.176 g, 11.57 mmol) was added in portions under Ar atmosphere and the resultant mixture was stirred at ambient temperature for 30 minutes. Compound 24 (4.5 g, 11.57 mmol) was added batch-wise to the above clear solution while stirring. The reaction mixture was stirred at room temperature for 2 hours. The reaction mixture was then concentrated under reduced pressure, and the resulting residue was purified directly by chromatography on SiO_2_ (isocratic elution with 1:6 EtOAc/petroleum ether) to provide the title compound (5.2 g, 9.62 mmol, 83% yield). Low resolution mass spectroscopy (LRMS): (ES, *m/z*): 540.20 [M+H]^+^. ^1^H NMR: (400 MHz, CDCl_3_, ppm): δ 8.01 (d, *J*=8.0 Hz, 2H), 7.93 (d, *J*=8.0 Hz, 2H), 7.43 (d, *J*=3.6 Hz, 1H), 7.22–7.37 (m, 4H), 6.80 (dd, *J*=6.0, 8.0 Hz, 1H), 6.61 (d, *J*=4.0 Hz, 1H), 5.76 (t, *J*=2.8 Hz, 1H), 4.76 (dd, *J*=3.6, 12.0 Hz, 1H), 4.63–4.69 (m, 2H), 2.76-2.86 (m, 2H), 2.47, 2.45 (2s, 6H).

*Step 5: Synthesis of (2R,3S,5R)-5-(4-amino-2-chloro-7H-pyrrolo[2,3-d]pyrimidin-7-yl)-2-(hydroxy-methyl)tetrahydrofuran-3-ol* **(Compound 26)**

Compound 25 (5 g, 9.25 mmol) was added to a 250-mL steel bomb and the medium cooled to −40 °C. Isopropanolic ammonia (isopropanol/liquid ammonia = 1/3, v/v, 150 mL) was then added. The medium was sealed, heated to 90 °C and stirred for 16 hours. The resulting mixture was cooled to room temperature and concentrated under reduced pressure. The residue was re-dissolved in MeOH (100 mL), followed by the addition of a solution of sodium methoxide in MeOH (1.0 M, 16.89 mL, 16.89 mmol) dropwise at 0 °C. The resulting mixture was heated to 25 °C and stirred for 2 hours. The mixture was neutralized by the addition of acetic acid (AcOH) (0.01 mL) and concentrated under reduced pressure. The residue was suspended in 1% MeOH/CH_2_Cl_2_ (150 mL), and after stirring for 20 minutes, a solid, white precipitation developed. The solid precipitation was collected by filtration, washed with CH_2_Cl_2_ and dried in a vacuum to provide the title compound (3.2 g, 6.74 mmol, 80% yield). LRMS: (ES, *m/z*): 285.20 [M+H]^+^. ^1^H NMR: (400 MHz, deuterated methanol [CD_3_OD], ppm): δ 7.31 (d, *J*=2.8 Hz, 1H), 6.58 (d, *J*=2.8 Hz, 1H), 6.45 (t, *J*=6.8 Hz, 1H), 4.52–4.53 (m, 1H), 3.98–4.01 (m, 1H), 3.81 (dd, *J*=2.4, 12.0 Hz, 1H), 3.73 (dd, *J* = 3.2, 12.0 Hz, 1H), 2.63–2.70 (m, 1H), 2.29–2.34 (m, 1H).

*Step 6: Synthesis of 7-((2R,4S,5R)-4-((tert-butyldimethylsilyl)oxy)-5-(((tert-butyldimethylsilyl)oxy)-methyl)tetrahydrofuran-2-yl)-2-chloro-7H-pyrrolo[2,3-d]pyrimidin-4-amine* **(Compound 27)**

TBSCl (3.05 g, 20.23 mmol) was added to a stirred solution of Compound 26 (3.2 g, 6.74 mmol) and imidazole (1.836 g, 27.0 mmol) in dry DMF (10 mL) under Ar atmosphere at room temperature. The resulting mixture was stirred overnight. The reaction mixture was diluted with EtOAc (100 mL), washed with water (2 x 20 mL), saturated aqueous NaHCO_3_ (2x 20 mL) and brine (2x 20 mL). The organic layer was dried over anhydrous Na_2_SO_4_, filtered, and concentrated under reduced pressure. The resulting residue was purified by chromatography on SiO_2_ (gradient elution 35% to 42% EtOAc/petroleum ether) to provide the title compound (2.4 g, 4.68 mmol, 69.3% yield). LRMS: (ES, m/z): 513.20 [M+H]^+^. ^1^H NMR: (400 MHz, CDCl_3_, ppm): δ 7.32 (s, 1H), 6.61 (t, *J*=6.8 Hz, 1H), 6.38 (s, 1H), 5.44 (br, 2H), 4.59–4.61 (m, 1H), 3.91–3.99 (m, 1H), 3.77–3.88 (m, 2H), 2.44-2.51 (m, 1H), 2.34–2.37 (m, 1H), 0.93-0.95 (m, 18H), 0.11–0.12 (m, 12H).

*Step 7: Synthesis of ((2R,3S,5R)-5-(4-amino-2-chloro-7H-pyrrolo[2,3-d]pyrimidin-7-yl)-3-((tert-butyldimethylsilyl)oxy)tetrahydrofuran-2-yl)methanol* **(Compound 28)**

To a stirred solution of Compound 27 (2.3 g, 4.48 mmol) in THF (40 mL) a pre-cooled solution of 1:1 TFA/water (20 mL) was added dropwise while stirring at 0 °C over 10 minutes. The resulting mixture was stirred for 2 hours at 0 °C. The reaction progress was monitored by TLC. The resulting solution was co-evaporated with toluene (3x 60 mL) while maintaining an internal temperature below 25 °C. The resulting residue was purified by chromatography on SiO_2_ (isocratic elution with 1:1 EtOAc/CH_2_Cl_2_) to provide the title compound (1.2 g, 3.01 mmol, 67.1% yield). LRMS: (ES, m/z): 399.20 [M+H]^+^. ^1^H NMR: (400 MHz, *d_6_*-DMSO, ppm): δ 7.53 (br, 2H), 7.35 (d, *J*=3.2 Hz, 1H), 6.60 (d, *J*=3.2 Hz, 1H), 6.35 (t, *J*=6.8 Hz, 1H), 4.95 (t, *J*=5.4 Hz, 1H), 4.51–4.54 (m, 1H), 3.73–3.79 (m, 1H), 3.46–3.55 (m, 2H), 2.54–2.59 (m, 1H), 2.16–2.20 (m, 1H), 0.90 (s, 9H), 0.11 (s, 6H).

*Step 8: Synthesis of (2S,3S,5R)-5-(4-amino-2-chloro-7H-pyrrolo[2,3-d]pyrimidin-7-yl)-3-((tert-butyldimethylsilyl)oxy)tetrahydrofuran-2-carbaldehyde* **(Compound 29)**

IBX (632 mg, 2.256 mmol) was added to a stirred solution of Compound 28 (600 mg, 1.504 mmol) in anhydrous DMSO (3 mL) and MeCN (15 mL) under Ar atmosphere . The mixture was stirred at room temperature for 2.5 hours. The reaction mixture was diluted with EtOAc (20 mL), and washed with brine (2x 10 mL), dried over anhydrous Na_2_SO_4_, and filtered. The filtrate was concentrated under reduced pressure to provide the title compound (700 mg, crude), which was used in the subsequent step without further purification. LRMS: (ES, m/z): 415.40 [M+H_2_O]^+^.

*Step 9: Synthesis of ((3S,5R)-5-(4-amino-2-chloro-7H-pyrrolo[2,3-d]pyrimidin-7-yl)-3-((tert-butyldimethylsilyl)oxy)tetrahydrofuran-2,2-diyl)dimethanol* **(Compound 30)**

To a stirred solution of Compound 29 (700 mg, crude) in 1,4-dioxane (28 mL) and water (7 mL), a formaldehyde solution (7 mL, 93 mmol) was added, followed by addition a 2N aqueous solution of NaOH (7 mL, 14 mmol) dropwise over 5 minutes. The resulting mixture was stirred at 25 °C for 3 hours. Next, the reaction mixture was neutralized by the addition of AcOH (0.5 mL), and the mixture was diluted with EtOAc (100 mL) and washed water (2x 20 mL), saturated aqueous NaHCO_3_ (20 mL), and brine (20 mL). The organic layer was dried over Na_2_SO_4_, filtered, and concentrated under reduced pressure. The resulting residue was re-dissolved in anhydrous EtOH (4 mL) and NaBH_4_ (133 mg, 3.53 mmol) was added under Ar atmosphere in portions at 0 °C. After stirring for 1 hour at 25 °C, the mixture was neutralized by the addition of AcOH (1.0 mL). The mixture was concentrated under reduced pressure, and the residue was diluted with CHCl_3_ (100 mL) and the washed with water (2x 20 mL) and brine (1x 20 mL). The organic layer was dried over Na_2_SO_4_, filtered, and concentrated under reduced pressure. The resulting residue was purified by chromatography on SiO_2_ (isocratic elution with 1:30 MeOH/CH_2_Cl_2_) to provide the title compound (150 mg, 0.350 mmol, two steps 19.83% yield). LRMS: (ES, m/z): 429.30 [M+H]^+^. ^1^H NMR: (300 MHz, *d_6_*-DMSO, ppm): δ 7.51 (br, 2H), 7.38 (d, *J*=3.9 Hz, 1H), 6.60 (d, *J*=3.6 Hz, 1H), 6.40 (t, *J*=6.9 Hz, 1H), 4.88 (t, *J*=5.5 Hz, 1H), 4.62 (dd, *J*=3.3, 5.5 Hz, 1H), 4.39 (t, *J*=5.7 Hz, 1H), 3.43–3.63 (m, 4H), 2.61-2.70 (m, 1H), 2.21–2.29 (m, 1H), 0.90 (s, 9H), 0.10 (s, 6H).

*Step 10: Synthesis of (2R,3S,5R)-5-(4-amino-2-chloro-7H-pyrrolo[2,3-d]pyrimidin-7-yl)-3-((tert-butyldimethylsilyl)oxy)-2-(hydroxymethyl)tetrahydrofuran-2-carbaldehyde* **(31)**

IBX (294 mg, 1.049 mmol) was added to a stirred solution of **30** (150 mg, 0.350 mmol) in dry MeCN (15 mL) under Ar atmosphere. The resulting mixture was stirred at 30 °C for 2 hours. The mixture was filtered and washed with CHCl_3_ (3x 15 mL). The filtrate was collected and then concentrated under reduced pressure to provide the title compound (200 mg, crude), which was used in the subsequent step without further purification. LRMS: (ES, m/z): 427.30 [M+H]^+^.

*Step 11: Synthesis of ((2R,3S,5R)-5-(4-amino-2-chloro-7H-pyrrolo[2,3-d]pyrimidin-7-yl)-3-((tert-butyldimethylsilyl)oxy)-2-ethynyltetrahydrofuran-2-yl)methanol* **(Compound 32)**

Compound 31 (200 mg, crude, 0.468 mmol) was dissolved in anhydrous MeOH (9 mL) followed by the addition of K_2_CO_3_ (162 mg, 1.171 mmol) at 0 °C under Ar atmosphere. A solution of dimethyl (1-diazo-2-oxopropyl)phosphonate (180 mg, 0.937 mmol) in MeOH (1 mL) was added to the suspension dropwise while stirring for 1 minute. The resulting mixture was heated to 30 °C and stirred for 16 hours. The reaction mixture was concentrated under reduced pressure, and the resulting residue was partitioned between EtOAc (30 mL) and water (10 mL). The organic layer was collected, washed with brine (2x 10 mL), dried over anhydrous Na_2_SO_4_, filtered, and concentrated under reduced pressure. The residue was then purified by preparative TLC (1:15 MeOH/CH_2_Cl_2_) to generate the title compound (60 mg, 0.136 mmol, two steps 29.1% yield). LRMS: (ES, m/z): 423.60 [M+H]^+^. ^1^H NMR: (400 MHz, *d_6_*-DMSO, ppm): δ 7.55 (br, 2H), 7.34 (d, *J*=3.6 Hz, 1H), 6.61 (d, *J*=3.6 Hz, 1H), 6.38 (t, *J*=6.4 Hz, 1H), 5.32 (t, *J*=6.0 Hz, 1H), 4.67 (t, *J*=6.0 Hz, 1H), 3.58–3.61 (m, 1H), 3.48–3.52 (m, 1H), 3.47 (s, 1H), 2.60–2.63 (m, 1H), 2.31–2.35 (m, 1H), 0.91 (s, 9H), 0.11 (d, *J* = 3.6Hz, 6H).

*Step 12: Synthesis of (2R,3S,5R)-5-(4-amino-2-chloro-7H-pyrrolo[2,3-d]pyrimidin-7-yl)-2-ethynyl-2-(hydroxymethyl)tetrahydrofuran-3-ol* **(Compound 12, MK-8527)**

A 1 M solution of TBAF in THF solution (0.28 mL, 0.284 mmol) was added to a stirred solution of Compound 32 (60 mg, 0.142 mmol) in THF (1 mL) dropwise while stirring at room temperature. The resulting solution was stirred at room temperature for 2 hours, and then the mixture was concentrated under reduced pressure. The residue was then purified by preparative HPLC [Prep-HPLC-011(Waters); Column: SunFire Prep C18 OBD Column, 5 mm, 19*150 mm; Mobile phase: water (with 10 mmol ammonium bicarbonate [NH_4_HCO_3_]) and MeCN; Detector: UV 254 and 220 nm] to provide **MK-8527** (31 mg, 0.100 mmol, 70.8% yield). LRMS: (ES, m/z): 309.00 [M+H]^+^. ^1^H NMR: (400 MHz, *d_6_*-DMSO, ppm): δ 7.54 (br, 2H), 7.33 (d, *J*=4.0 Hz, 1H), 6.60 (d, *J*=3.6 Hz, 1H), 6.41 (t, *J*=6.4 Hz, 1H), 5.52–5.53 (m, 1H), 5.26 (t, *J*=6.0 Hz, 1H), 4.47–4.48 (m, 1H), 3.61 (dd, *J*=5.2, 11.6 Hz, 1H), 3.53 (dd, *J*=6.2, 11.8 Hz, 1H), 3.48 (s, 1H), 2.41–2.50 (m, 1H), 2.32–2.38 (m, 1H).

**Compound Synthesis Examples**

**Synthesis of Compound 1** *(2R,3S,5R)-5-(6-amino-2-chloro-9H-purin-9-yl)-2-ethynyl-2-(hydroxymethyl)-tetrahydrofuran-3-ol* **(Compound 1)**

**Fig B.** **Synthesis of Compound 1**.^a^

^a^Reagents and conditions: (a) RedAl, CH_2_Cl_2_, toluene, –78 °C; (b) acetic anhydride (Ac_2_O), dimethylamino pyridine (DMAP), CH_2_Cl_2_; (c) 2,6-dichloro-9H-purine, benzene sulfonic acid (BSA), trimethylsilyl trifluoromethanesulfonate (TMSOTf), MeCN, 80 °C; (d) NH_3_, MeOH.

(2*R*,3*S*)-2-ethynyl-2-(((4-methylbenzoyl)oxy)methyl)-5-oxotetrahydrofuran-3-yl 4-methylbenzoate was prepared in 11 steps according to the experimental procedure described in: McLaughlin, M., *et al*. *Org. Lett.* **2017**, *19*, 926. [1]

*Step 1: Synthesis of (2R,3S)-2-ethynyl-5-hydroxy-2-(((4-methylbenzoyl)oxy)methyl)-tetrahydrofuran-3-yl 4-methylbenzoate*

To a stirred solution of methyl (2*R*,3*S*)-2-ethynyl-2-(((4-methylbenzoyl)oxy)methyl)-5-oxotetrahydrofuran-3-yl 4-methylbenzoate (1160 mg, 2.96 mmol) in anhydrous toluene (30 mL) and CH_2_Cl_2_ (6 mL) under Ar atmosphere in a 100-mL 3-necked round-bottom flask, toluene solution of bis(2-methoxyethoxy)aluminum(III) sodium hydride (RedAl, 70% w/w, 0.598 g, 2.96 mmol) was added dropwise while stirring at –78 °C over 3 minutes. The resulting solution was stirred at the same temperature for 90 minutes. The reaction was quenched by the addition of AcOH (1.7 mL), and then 1N HCl (30 mL) was added and the mixture was extracted with EtOAc (3x 30 mL). The combined organic fractions were washed with brine (2x 30 mL), dried over anhydrous Na_2_SO_4_, filtered and concentrated under reduced pressure. The resulting residue was purified by chromatography on SiO_2_ (isocratic elution with 15:85 EtOAc/petroleum ether) to provide the title compound (925 mg, 2.345 mmol, 79% yield). ^1^H NMR: (300 MHz, CDCl_3_, ppm): δ 7.92–-8.00 (m, 4H), 7.19–7.27 (m, 4H), 5.74–5.84 (m, 1H), 5.65–5.70 (m, 1H), 4.68 (s, 1H), 4.54 (dd, *J*=11.4 Hz, 35.4 Hz, 1H), 2.49–2.61 (m, 2.5H), 2.36–2.42 (m, 6.5H).

*Step 2: Synthesis of (2R,3S)-5-acetoxy-2-ethynyl-2-(((4-methylbenzoyl)oxy)methyl)-tetrahydrofuran-3-yl 4-methylbenzoate*

To a stirred solution of (2*R*,3*S*)-2-ethynyl-5-hydroxy-2-(((4-methylbenzoyl)oxy)methyl)-tetrahydrofuran-3-yl 4-methylbenzoate (925 mg, 2.345 mmol) in CH_2_Cl_2_ (30 mL) under Ar atmosphere in a 100-mL 3-necked round-bottom flask, DMAP (430 mg, 3.52 mmol) was added, followed by the addition of a solution of Ac_2_O (0.332 mL, 3.52 mmol) in CH_2_Cl_2_ (3 mL) dropwise while stirring at 0 ^°^C. The resulting solution was stirred at 0 ^°^C for 1 hour and the reaction was then quenched with water (30 mL) and extracted with CH_2_Cl_2_ (3x 30 mL). The combined organic layers were washed with brine (2x 30 mL), dried over anhydrous Na_2_SO_4_, filtered, and concentrated under reduced pressure. The resulting residue was purified by chromatography on SiO_2_ (isocratic elution with 1:9 EtOAc/petroleum ether) to provide the title compound (1 g, 2.291 mmol, 98% yield). ^1^H NMR: (300 MHz, CDCl_3_, ppm): δ 7.90–8.10 (m, 4H), 7.19–7.27 (m, 4H), 6.45–6.50 (m, 1H), 5.84 (t, *J*=7.5 Hz, 1H), 4.72–4.75 (d, *J*=11.7 Hz, 1H), 4.50–4.61 (m, 1H), 2.64–2.78 (m, 3H), 2.40–2.43 (m, 6H), 1.90 (s, 3H).

*Step 3: Synthesis of (2R,3S,5R)-5-(2,6-dichloro-9H-purin-9-yl)-2-ethynyl-2-(((4-methylbenzoyl)oxy)-methyl)tetrahydrofuran-3-yl 4-methylbenzoate*

BSA (559 mg, 2.75 mmol) was added in one portion to a stirred suspension of 2,6-dichloro-9H-purine (113 mg, 0.596 mmol) in MeCN (8 mL) under Ar atmosphere in a 100-mL 3-necked round-bottom flask. The resulting mixture was refluxed for 2 hours. The resulting clear solution was cooled to 0 ^°^C, and then a solution of (2*R*,3*S*)-5-acetoxy-2-ethynyl-2-(((4-methylbenzoyl)oxy)methyl)tetrahydrofuran-3-yl 4-methylbenzoate (200 mg, 0.458 mmol) in MeCN (1 mL) was added, followed by TMSOTf (204 mg, 0.916 mmol). The mixture was refluxed overnight. The resulting mixture was cooled, quenched by the addition of saturated aqueous NaHCO_3_ (20 mL) and the mixture was extracted with EtOAc (2x 20 mL). The combined organics were washed with brine (20 mL), dried over anhydrous Na_2_SO_4_, filtered, and concentrated under reduced pressure. The resulting residue was purified by chromatography on SiO_2_ (isocratic elution with 1:2 EtOAc/petroleum ether) to provide the title compound (163 mg, 0.288 mmol, 62.9% yield). ^1^H NMR: (300 MHz, CDCl_3_, ppm): δ 8.24 (s, 1H), 8.03 (d, *J*=8.1 Hz, 2H), 7.83 (d, *J*=8.1 Hz, 2H), 7.31 (d, *J*=8.1 Hz, 2H), 7.21 (d, *J*=8.1 Hz, 2H), 6.59 (t, *J*=6.3 Hz, 1H), 6.07 (t, *J*=6.6 Hz, 1H), 4.92 ( d, *J*=12.0 Hz, 1H), 4.64 (d, *J*=12 Hz, 1H), 3.16–3.25 (m, 1H), 2.95–3.04 (m, 1H), 2.74 (s, 1H), 2.46 (s, 3H), 2.42 (s, 3H).

*Step 4: Synthesis of (2R,3S,5R)-5-(6-amino-2-chloro-9H-purin-9-yl)-2-ethynyl-2-(hydroxymethyl)-tetrahydrofuran-3-ol*

A solution of *(2R,3S,5R)-5-(2,6-dichloro-9H-purin-9-yl)-2-ethynyl-2-(((4-methylbenzoyl)oxy)-methyl)tetrahydrofuran-3-yl 4-methylbenzoate* (80 mg, 0.141 mmol) in saturated methanolic NH_3_ (3 mL) was placed into a 10-mL sealed tube. The reaction mixture was heated to 80 °C for 3 hours. The mixture was cooled and the solvent removed under reduced pressure. The residue was then purified by preparative HPLC [Column: Xbrige RP18 19 x 150 mm; Mobile phase: water (with 0.05% NH_3_) and MeCN; Detector: UV 254 and 220 nm] to generate the title compound (31 mg, 0.100 mmol, 70.8% yield). LRMS: (ES, m/z): 310.0 [M+H]^+^. ^1^H NMR: (300 MHz, CD_3_OD, ppm): δ 8.34 (s, 1H), 7.83 (br, 2H), 6.27 (dd, *J*=5.3, 7.1 Hz, 1H), 5.57 (d, *J* =5.7 Hz, 1H), 5.30 (t, *J*=6.3 Hz, 1H), 4.55 (dd, *J*=6.6, 12.3 Hz, 1H), 3.65 (dd, *J*=5.6, 12 Hz, 1H), 3.56 (dd, *J*=6.7, 12 Hz, 1H), 3.51 (s, 1H), 2.66–2.74 (m, 1H), 2.39–2.46 (m, 1H).

**Synthesis of Compound 2** *(2R,3S,5R)-5-(6-amino-2-methyl-9H-purin-9-yl)-2-ethynyl-2-(hydroxymethyl)-tetrahydrofuran-3-ol* **(Compound 2)**

Compound 2 was prepared according to a similar synthetic protocol as described for the synthesis of Compound 1. LRMS: (ES, m/z): 290.05 [M+H]^+^. ^1^H NMR: (300 MHz, *d_6_*-DMSO, *ppm*): δ 8.22 (s, 1H), 7.22 (br, 2H), 6.35 (t, *J*=6.6 Hz, 1H), 5.85–5.87 (m, 1H), 5.56 (s, 1H), 4.48–4.56 (m, 1H), 3.67–3.72 (m, 1H), 3.55–3.61 (m, 1H), 3.51 (s, 1H), 2.70–2.79 (m, 1H), 2.32–2.38 (m, 4H).

**Synthesis of Compound 3** *(2R,3S,5R)-5-(6-amino-2-(trifluoromethyl)-9H-purin-9-yl)-2-ethynyl-2-(hydroxymethyl)tetrahydrofuran-3-ol* **(Compound 3)**

Compound 3 was prepared according to a similar synthetic protocol as described for the synthesis of Compound 1. LRMS: (ES, m/z): 344.10 [M+H]^+^. ^1^H NMR: (400 MHz, CD_3_OD, ppm): δ 8.46 (s, 1H), 6.52–6.49 (m, 1H), 4.84 (t, *J*=7.2 Hz, 1H), 3.85 (dd, *J*=12, 37.6 Hz, 2H), 3.11 (s, 1H), 2.89–2.84 (m, 1H), 2.71–2.63 (m, 1H). F-NMR (376 MHz, CD_3_OD, ppm): *δ* -71.11; MS (ES, *m/z*): 344.10 [M+H]^+^.

**Synthesis of Compound 4** *(2R,3S,5R)-5-(6-(cyclopropylamino)-2-fluoro-9H-purin-9-yl)-2-ethynyl-2-(hydroxymethyl)tetrahydrofuran-3-ol* **(Compound 4)**

Compound 4 was prepared according to a similar synthetic protocol as described for the synthesis of Compound 1. LRMS: (ES, m/z): 356.1 [M+Na]^+^. ^1^H NMR: (300 MHz, *d_6_*-DMSO, *ppm*): δ 8.57 (br, 1H), 8.31 (s, 1H), 6.25 (dd, *J*=5.1, 6.9 Hz, 1H), 5.57 (s, 1H), 5.29 (s, 1H), 4.57 (t, *J*=5.7 Hz, 1H), 3.65 (d, *J*=11.1 Hz, 1H), 3.55 (d, *J*=11.1 Hz, 1H), 3.51 (s, 1H), 2.85–2.96 (m, 1H), 2.66–2.74 (m, 1H), 2.38–2.45 (m, 1H), 0.71–0.78 (m, 2H), 0.63–0.67 (m, 2H).

**Synthesis of Compound 5** *(2R,3S,5R)-5-(4-amino-7H-pyrrolo[2,3-d]pyrimidin-7-yl)-2-ethynyl-2-(hydroxymethyl)tetrahydrofuran-3-ol* **(Compound 5)**

Compound 5 was prepared according to a similar synthetic protocol as described for the synthesis of MK-8527. LRMS: (ES, m/z): 275.20 [M+H]^+^. ^1^H NMR: (300 MHz, *d_6_*-DMSO, *ppm*): δ 8.05 (s, 1H), 7.31 (d, *J*=3.6 Hz, 1H), 7.03 (brs, 2H), 6.58 (d, *J*=3.6 Hz, 1H), 6.50 (t, *J*=6.3 Hz, 1H), 5.40–5.49 (m, 2H), 4.49 (dd, *J*=6.0, 11.4 Hz, 1H), 3.51–3.66 (m, 2H), 3.46 (s, 1H), 2.55–2.59 (m, 1H), 2.28–2.39 (m, 1H).

**Synthesis of Compound 6** *(2R,3S,5R)-5-(4-amino-6-methyl-7H-pyrrolo[2,3-d]pyrimidin-7-yl)-2-ethynyl-2-(hydroxymethyl)tetrahydrofuran-3-ol* **(Compound 6)**

Compound 6 was prepared according to a similar synthetic protocol as described for the synthesis of MK-8527. LRMS: (ES, m/z): 289.3 [M+H]^+^. ^1^H NMR: (600 MHz, *d_6_*-DMSO, *ppm*): δ 7.98 (s, 1H), 6.93 (s, 2H), 6.43 (t, *J*=7.2 Hz, 1H), 6.31 (d, *J*=1.0 Hz, 1H), 5.88 (dd, *J*=8.3, 4.5 Hz, 1H), 5.45 (d, *J*=5.6 Hz, 1H), 4.58 (dt, *J*=7.4, 5.6 Hz, 1H), 3.70 (dd, *J*=11.9, 4.5 Hz, 1H), 3.56 (dd, *J*=11.9, 8.4 Hz, 1H), 3.49 (s, 1H), 2.89 (dt, *J*=13.2, 7.3 Hz, 1H), 2.43–2.40 (m, 3H), 2.24 (ddd, *J*=13.1, 7.7, 5.5 Hz, 1H).

**Synthesis of Compound 7** *(2R,3S,5R)- 5-(4-amino-5-methyl-7H-pyrrolo[2,3-d]pyrimidin-7-yl)-2-ethynyl-2-(hydroxymethyl)tetrahydrofuran-3-ol* **(Compound 7)**

Compound 7 was prepared according to a similar synthetic protocol as described for the synthesis of MK-8527. LRMS: (ES, m/z): 289.05 [M+H]^+^. ^1^H NMR: (300 MHz, *d_6_*-DMSO, ppm): δ 8.00 (s, 1H), 7.06 (s, 1H), 6.58 (br, 2H), 6.48 (t, *J*=6.60 Hz, 1H), 5.45 (d, *J*=5.1 Hz, 1H), 5.38 (t, *J*=6.0 Hz, 1H), 4.46 (q, *J*=5.4 Hz, 1H), 3.50-3.64 (m, 2H), 3.44 (s, 1H), 2.44–2.54 (m, 1H), 2.33 (s, 3H), 2.24–2.32 (m, 1H).

**Synthesis of Compound 8** *(2R,3S,5R)-5-(4-amino-5-methyl-7H-pyrrolo[2,3-d]pyrimidin-7-yl)-2-ethynyl-2-(hydroxymethyl)tetrahydrofuran-3-ol* **(Compound 8)**

Compound 8 was prepared according to a similar synthetic protocol as described for the synthesis of MK-8527. LRMS: (ES, m/z): 308.72 [M+H]^+^. ^1^H NMR: (400 MHz, *d_6_*-DMSO, ppm): δ 8.10 (s, 1H), 7.54 (s, 1H), 6.88 (br, 2H), 6.52 (t, *J*=6.4 Hz, 1H), 5.39–5.52 (m, 2H), 4.48 (t, *J*=6.6 Hz, 1H), 3.59 (dd, *J*=12.0, 28.8 Hz, 2H), 3.48 (s, 1H), 2.47–2.54 (m, 1H), 2.31–2.37 (m, 1H).

**Synthesis of Compound 9** *(2R,3S,5R)-5-(4-amino-5-fluoro-7H-pyrrolo[2,3-d]pyrimidin-7-yl)-2-ethynyl-2-(hydroxymethyl)tetrahydrofuran-3-ol* **(Compound 9)**

**Fig C.** **Synthesis of Compound 9**.^a^

^a^Reagents and conditions: (a) phenyl boronic acid, Pd_2_(Ph_3_P)_4_, Na_2_CO_3_, 1,4-dioxane; (b) TBAF, THF.

*((2R,3S,5R)-5-(4-amino-5-iodo-7H-pyrrolo[2,3-d]pyrimidin-7-yl)-3-((tert-butyldimethyl-silyl)oxy)-2-ethynyltetrahydrofuran-2-yl)methanol* was prepared according to a similar synthetic protocol as described for the synthesis of MK-8527. LRMS: (ES, m/z): 515.10 [M+H]^+^. ^1^H NMR: (400 MHz, CDCl_3_, ppm): 8.22 (s, 1H), 7.12 (s, 1H), 6.25 (dd, *J*=8.8, 5.6 Hz, 1H), 5.84 (brs, 2H), 4.71 (dd, *J*=5.6, 2 Hz,1H), 4.01 (d, *J*=12.4 Hz, 1H), 3.80 (d, *J*=16 Hz, 1H), 3.17–3.10 (m, 1H), 2.59 (s, 1H), 2.27–2.21 (m, 1H), 0.91 (s, 9H), 0.11 (s, 6H).

*Step 1: Synthesis of ((2R,3S,5R)-5-(4-amino-5-phenyl-7H-pyrrolo[2,3-d]pyrimidin-7-yl)-3-((tert-butyldimethylsilyl)oxy)-2-ethynyltetrahydrofuran-2-yl)methanol*

Pd_2_(Ph_3_P)_4_ (11.68 mg, 10.11 µmol) was added to ((2*R*,3*S*,5*R*)-5-(4-amino-5-iodo-7*H*-pyrrolo[2,3-d]pyrimidin-7-yl)-3-((tert-butyldimethylsilyl)oxy)-2-ethynyltetrahydrofuran-2-yl)methanol (52 mg, 0.101 mmol), phenylboronic acid (18.49 mg, 0.152 mmol), and Na_2_CO_3_ (0.758 mL, 1.516 mmol) in 1,4-dioxane (2 mL) at 20 °C. This mixture was gassed under Ar atmosphere. The resulting mixture was stirred for 3 hours at 100 °C under Ar atmosphere. The reaction mixture was allowed to cool to room temperature, concentrated under reduced pressure, and the resulting residue was purified by preparative TLC (elution with 5% MeOH/CH_2_Cl_2_) to generate the title compound (20 mg, 0.043 mmol, 42.6% yield). LRMS: (ES, *m/z*): 465.3 [M + H]^+ 1^. ^1^H NMR: (300 MHz, CDCl_3_, ppm): 8.33 (s, 1H), 7.55–7.45 (m, 5H), 7.03 (s, 1H), 6.34 (dd, *J*=9, 5.4 Hz, 1H), 5.48 (brs, 2H), 4.75 (d, *J*=3.6 Hz, 1H), 4.16-4.01 (m, 2H), 3.84–3.78 (m, 1H), 3.24–3.21 (m, 1H), 2.59 (s, 1H), 2.36–2.28 (m, 1H), 0.91 (s, 9H), 0.12 (s, 6H).

*Step 2: Synthesis of (2R,3S,5R)-5-(4-amino-5-fluoro-7H-pyrrolo[2,3-d]pyrimidin-7-yl)-2-ethynyl-2-(hydroxymethyl)tetrahydrofuran-3-ol* **(9)**

A 1 M THF solution of TBAF (0.043 mL, 0.043 mmol) was added to ((2*R*,3*S*,5*R*)-5-(4-amino-5-phenyl-7*H*-pyrrolo[2,3-d]pyrimidin-7-yl)-3-((tert-butyldimethylsilyl)oxy)-2-ethynyltetrahydrofuran-2-yl)methanol (20 mg, 0.043 mmol) in THF (2 mL) at 0 °C under Ar atmosphere, and the mixture was stirred at 25 °C for 1 hour. Solvent was removed under reduced pressure, and the resulting residue was purified by preparative TLC (elution with 10% MeOH/CH_2_Cl_2_) to provide 10 mg of impure material, which was further purified by preparative HPLC [Column: XBridge RP18 19x150 mm; Mobile phase: water (with 0.05% NH_4_HCO_3_) and MeCN; Detector: UV 254 and 220 nm] to generate the title compound (3.2 mg, 9.13 µmol, 21.22% yield). LRMS: (ES, *m/z*): 350.9 [M + H]^+^.  ^1^H NMR: (300 MHz, d_6_-DMSO, ppm): 8.16 (s, 1H), 7.50–7.40 (m, 5H), 7.37 (t, *J*=4.5 Hz, 1H), 6.61 (t, *J*=6.3 Hz, 1H), 6.15 (brs, 2H), 5.52 (d, *J*=5.4 Hz, 1H), 5.41 (t, *J*=6 Hz, 1H), 4.53 (d, *J*=6 Hz, 1H), 3.67–3.55 (m, 2H), 3.55 (s, 1H), 2.64–2.50 (m, 1H), 2.42–2.36 (m, 1H).

**Synthesis of Compound 10** *(2R,3S,5R)-5-(4-amino-5-(1H-pyrazol-5-yl)-7H-pyrrolo[2,3-d]pyrimidin-7-yl)-2-ethynyl-2-(hydroxymethyl)tetrahydrofuran-3-ol* **(Compound 10)**

Compound 10 was prepared according to a similar synthetic protocol as described for the synthesis of Compound 9. LRMS: (ES, m/z): 341.1 [M+H]^+^. ^1^H NMR: (300 MHz, CD_3_OD, *ppm*): δ 8.04 (s, 1H), 7.76 (s, 1H), 7.65 (d, *J*=2.4 Hz, 1H), 6.64–6.58 (m, 2H), 4.70 (t, *J*=6.9 Hz, 1H), 3.90–3.76 (m, 2H), 3.09 (s, 1H), 2.78–2.66 (m, 1H), 2.60–2.51 (m, 1H).

**Synthesis of Compound 11** *(2R,3S,5R)-5-(4-amino-2-fluoro-7H-pyrrolo[2,3-d]pyrimidin-7-yl)-2-ethynyl-2-(hydroxymethyl)tetrahydrofuran-3-ol* **(Compound 11)**

Compound 11 was prepared according to a similar synthetic protocol as described for the synthesis of MK-8527. LRMS: (ES, m/z): 315.15 [M+H]^+^. ^1^H NMR: (400 MHz, *d_6_*-DMSO, ppm): δ 7.57 (brs, 2H), 7.28 (d, *J*=3.6 Hz, 1H), 6.61 (d, *J*=3.6 Hz, 1H), 6.35 (t, *J*=6.4 Hz, 1H), 5.51 (d, *J*=5.6 Hz, 1H), 5.27 (t, *J*=6.0 Hz, 1H), 4.48 (dd, *J*=6.4, 12.0 Hz, 1H), 3.61 (dd, *J*=5.6, 11.6 Hz, 1H), 3.53 (dd, *J*=6.4, 12.0 Hz, 1H), 3.48 (s, 1H), 2.50–2.46 (m, 1H), 2.37–2.31 (m, 1H). ^19^F NMR: (376 MHz, *d_6_*-DMSO, ppm): δ −53.67 (s, 1F).

**Synthesis of Compound 13** *(2R,3S,5R)-5-(4-amino-2-chloro-5-fluoro-7H-pyrrolo[2,3-d]pyrimidin-7-yl)-2-ethynyl-2-(hydroxymethyl)tetrahydrofuran-3-ol* **(Compound 13)**

Compound 13 was prepared according to a similar synthetic protocol as described for the synthesis of MK-8527. LRMS: (ES, m/z): 327.00 [M+H]^+^. ^1^H NMR: (300 MHz, *d_6_*-DMSO, ppm): δ 7.68 (brs, 2H), 7.32 (d, *J*=2.0 Hz, 1H), 6.44 (t, *J*=5.8 Hz, 1H), 5.52 (d, *J*=5.6 Hz, 1H), 5.27 (t, *J*=6.0 Hz, 1H), 4.44 (q, *J*=6.4 Hz, 1H), 3.60 (q, *J*=6.0 Hz, 1H), 3.53 (q, *J*=6.4 Hz, 1H), 3.48 (s, 1H), 2.48–2.41 (m, 1H), 2.37–2.30 (m, 1H). ^19^F NMR: (282 MHz, *d_6_*-DMSO): δ (ppm) −166.67 (s, 1F).

**Synthesis of Compound 14** *(2R,3S,5R)-5-(2,4-diamino-7H-pyrrolo[2,3-d]pyrimidin-7-yl)-2-ethynyl-2-(hydroxymethyl)tetrahydrofuran-3-ol* **(Compound 14)**

Compound 14 was prepared according to a similar synthetic protocol as described for the synthesis of MK-8527. LRMS: (ES, m/z): 290.3 [M+H]^+^. ^1^H NMR: (400 MHz, CDCl_3_, ppm): δ 6.85 (d, *J*=3.6 Hz, 1H), 6.55 (s, 2H), 6.36 (d, *J*=4.0 Hz, 2H), 5.56 (s, 2H), 5.48–5.43 (m, 2H), 4.42 (q, *J*=5.6 Hz, 1H), 3.61–3.49 (m, 2H), 3.44 (s, 1H), 2.47–2.40 (m, 1H), 2.26–2.21 (m, 1H).

**Synthesis of Compound 15** *(2R,3S,5R)-5-(7-amino-3H-[1,2,3]triazolo[4,5-d]pyrimidin-3-yl)-2-ethynyl-2-(hydroxymethyl)tetrahydrofuran-3-ol* **(Compound 15)**

Compound 15 was prepared according to a similar synthetic protocol as described for the synthesis of MK-8527. LRMS: (ES, m/z): 277.2 [M+H]^+^. ^1^H NMR: (600 MHz, *d_6_*-DMSO, *ppm*): δ 8.48 (s, 1H), 8.32 (s, 1H), 8.14 (s, 1H), 6.59 (dd, *J*=7.7, 4.1 Hz, 1H), 5.60 (d, *J*=5.6 Hz, 1H), 5.08 (dd, *J*=7.0, 5.8 Hz, 1H), 4.78 (q, *J*=7.0 Hz, 1H), 3.61 (dd, *J*=11.9, 5.7 Hz, 1H), 3.50 (s, 1H), 3.48 (dd, *J*=11.9, 7.2 Hz, 1H), 3.03 (ddd, *J*= 13.2, 6.7, 4.1 Hz, 1H), 2.58 (dt, *J*=13.2, 7.7 Hz, 1H).

**Synthesis of Compound 16** *(2R,3S,5R)-5-(4-aminopyrrolo[2,1-f][1,2,4]triazin-7-yl)-2-ethynyl-2-(hydroxymethyl)tetrahydrofuran-3-ol* **(Compound 16)**

**Fig D.** **Synthesis of Compound 16.^a^**

**^a^**Reagents and conditions: (a) 4-(methylthio)pyrrolo-[2,1-f][1,2,4]triazine, lithium diisopropylamide (LDA), THF −78 °C to −50 °C; (b) triethylsilane (Et_3_SIH), BF_3_•Et_2_O; (c) NH_3_, isopropanol; (d) palladium hydroxide on carbon (Pd(OH)_2_/C), AcOH; (e) TIPDSCl, imidazole, DMF; (f) *O*-phenyl carbonochloridothioate, DMAP, CH_2_Cl_2_; (g) azobisisobutyronitrile (AIBN), tributyltin hydride (Bu_3_SnH), toluene; (h) TBAF, THF; (i) TBDMSCl, imidazole, DMF; (j) TFA/THF/H_2_O (1:4:1), 0 °C; (k) IBX, MeCN, 80 °C; (l) 1. formaldehyde, NaOH, 2. NaBH_4_, EtOH; (m) IBX, MeCN, 30 °C; (n) Bestmann−Ohira Reagent, K_2_CO_3,_ MeOH; (o) TBAF, THF.

*Step 1: Synthesis of (3R,4R,5R)-3,4-bis(benzyloxy)-5-((benzyloxy)methyl)-2-(4-(methylthio)-pyrrolo[2,1-f][1,2,4]-triazin-7-yl)tetrahydrofuran-2-ol*

Lithium diisopropylamide (LDA) (7.5 ml, 15.0 mmol) was added to a solution of 4-(methylthio)pyrrolo[2,1-f][1,2,4]triazine (1.65 g, 9.99 mmol) in THF (30 mL) at –78 ºC under Ar atmosphere and the resulting mixture was stirred for 30 minutes at –78 ºC. After that, (3*R*,4*R*,5*R*)-3,4-bis(benzyloxy)-5-((benzyloxy)methyl)-dihydrofuran-2(3H)-one (4.18 g, 9.99 mmol) in THF (10 mL) was added at –78 ºC under Ar atmosphere and stirred for 2 hours at –78 to –50 ºC. The reaction was quenched by addition of aqueous NH_4_Cl (10 mL), and then extracted with EtOAc (300 mL). The organic layer was washed with 3x 50 mL brine, dried over Na_2_SO_4_ and concentrated under reduced pressure. The resulting residue was purified by chromatography on SiO_2_ (isocratic elution with 1:5 EtOAc/petroleum ether) to provide the title compound (3.5 g, 6.00 mmol, 60.0% yield). LRMS: (ES, m/z): 584.2 [M+H]^+^.

*Step 2: Synthesis of 7-((2S,3S,4R,5R)-3,4-bis(benzyloxy)-5-((benzyloxy)methyl)tetrahydrofuran-2-yl)-4-(methylthio)-pyrrolo[2,1-f][1,2,4]triazine*

Triethylsilane (2.390 g, 20.56 mmol) and trifluoroborane (0.697 g, 10.28 mmol) were added to a solution of (3*R*,4*R*,5*R*)-3,4-bis(benzyloxy)-5-((benzyloxy)methyl)-2-(4-(methylthio)pyrrolo[2,1-f][1,2,4]-triazin-7-yl)tetrahydrofuran-2-ol (3.00 g, 5.14 mmol) in CH_2_Cl_2_ (30 mL) at 0 ºC under Ar atmosphere. The resulting solution was stirred for 30 minutes at 0 ºC and then quenched by additional of aqueous NaHCO_3_ (20 mL) and extracted with CH_2_Cl_2_ (200 mL). The organic layer was washed with brine (3x 50 mL), dried over Na_2_SO_4_ and concentrated under reduced pressure. The resulting residue was purified by chromatography on SiO_2_ (isocratic elution with 1:10 EtOAc/petroleum ether) to provide the title compound (2.0 g, 3.52 mmol, 68.5% yield). LRMS: (ES, m/z): 568.4 [M+H]^+^. ^1^H NMR: (300 MHz, CDCl_3_, ppm): δ 8.21 (s, 1H), 7.23–7.36 (m, 15H), 6.81 (d, *J*=4.5 Hz, 1H), 6.70 (d, *J*=4.5 Hz, 1H), 5.69 (d, *J*=4.2 Hz, 1H), 4.37–4.62 (m, 7H), 4.22–4.26 (m, 1H), 4.10–4.13 (m, 1H), 3.66–3.67 (m, 1H), 3.62–3.63 (m, 1H), 2.75 (s, 3H).

*Step 3: Synthesis of 7-((2S,3S,4R,5R)-3,4-bis(benzyloxy)-5-((benzyloxy)methyl)tetrahydrofuran-2-yl)pyrrolo[2,1-f][1,2,4]triazin-4-amine*

A solution of 7-((2*S*,3*S*,4*R*,5*R*)-3,4-bis(benzyloxy)-5-((benzyloxy)methyl)tetrahydrofuran-2-yl)-4-(methylthio)-pyrrolo[2,1-f][1,2,4]triazine (4.6 g, 8.10 mmol) in isopropanol (5 mL) was flushed with NH_3_ (1350 mg, 79 mmol) at –40 ºC for 1 hour in sealed tube. The resulting mixture was stirred for 16 hours at 80 ºC, and then concentrated to provide the title compound (3.0 g, 5.59 mmol, 69.0% yield), which was used in the subsequent step without further purification. LRMS: (ES, m/z): 537.3 [M+H]^+^. ^1^H NMR: (300 MHz, CDCl_3_, ppm): δ (300 MHz, *d*_6_-DMSO, ppm): δ 7.80 (s, 1H), 7.69 (s, 2H), 7.24–7.36 (m, 15H), 6.88 (d, *J*=4.5 Hz, 1H), 6.80 (d, *J*=4.2 Hz, 1H), 5.44 (d, *J*=5.4 Hz, 1H), 4.49–4.62 (m, 6H), 4.31 (t, *J*=5.1 Hz, 1H), 4.15–4.18 (m, 1H), 4.09–4.12 (m, 1H), 3.29–3.67 (m, 2H).

*Step 4: Synthesis of (2S,3R,4S,5R)-2-(4-aminopyrrolo[2,1-f][1,2,4]triazin-7-yl)-5-(hydroxymethyl)-tetrahydrofuran-3,4-diol*

Pd(OH)_2_/C (10 mol%, 100 mg, 0.130 mmol) was added to a solution of 7-((2*S*,3*S*,4*R*,5*R*)-3,4-bis(benzyloxy)-5-((benzyloxy)methyl)tetrahydrofuran-2-yl)pyrrolo[2,1-f][1,2,4]triazin-4-amine (100 mg, 0.186 mmol) in AcOH (2 mL) under H_2_ at 25 ºC for 4 hours. The mixture was filtered through a Celite pad, and the filtrate was evaporated. The resulting residue was purified by chromatography on SiO_2_ (isocratic elution with 1:8 MeOH/CH_2_Cl_2_) to provide the title compound (40 mg, 0.150 mmol, 81% yield) as colorless foam. LRMS: (ES, m/z): 267.2 [M+H]. ^1^H NMR: (300 MHz, DMSO, ppm): δ 7.79 (s, 1H), 7.77 (s, 2H), 6.84 (d, *J*=4.2 Hz, 1H), 6.68 (d, *J*=4.2 Hz, 1H), 5.10 (d, *J*=6.6 Hz, 1H), 4.95 (d, *J*=6.6 Hz, 1H), 4.84 (d, *J*=4.8 Hz, 1H), 4.76 (t, *J*=5.4 Hz, 1H), 4.20–4.25 (m, 1H), 3.94 (d, *J*=4.8 Hz, 1H), 3.78 (d, *J*=4.2 Hz, 1H), 3.41–3.64 (m, 2H).

*Step 5: Synthesis of (6aR,8S,9S,9aS)-8-(4-aminopyrrolo[2,1-f][1,2,4]triazin-7-yl)-2,2,4,4-tetraisopropyl-tetrahydro-6H-furo[3,2-f][1,3,5,2,4]trioxadisilocin-9-ol*

(2*S*,3*R*,4*S*,5*R*)-2-(4-aminopyrrolo[2,1-f][1,2,4]triazin-7-yl)-5-(hydroxymethyl)-tetrahydrofuran-3,4-diol (790 mg, 2.97 mmol) and imidazole (606 mg, 8.90 mmol) were dissolved in DMF (20 mL) under Ar atmosphere. 1,3-dichloro-1,1,3,3-tetraisopropyldisiloxane (936 mg, 2.97 mmol) was added dropwise at 0 ºC. After the resulting mixture was stirred for 5 hours at 25 ºC under Ar atmosphere, water (10 mL) was added and the resulting mixture was diluted with EtOAc (400 mL). The organic phase was washed with water (3x 60 mL) and brine (60 mL), dried over Na_2_SO_4_, and concentrated under reduced pressure. The resulting residue was purified by chromatography on SiO_2_ (isocratic elution with 1:20 MeOH/CH_2_Cl_2_) to provide the title compound (1.0 g, 1.966 mmol, 66.2% yield). LRMS: (ES, m/z): 509.3 [M+H]. ^1^H NMR: (400 MHz, DMSO, ppm): δ 7.81 (s, 1H), 7.70 (s, 2H), 6.82 (d, *J*=4.8 Hz, 1H), 6.61 (d, *J*=4.4 Hz, 1H), 5.20 (d, *J*=4.8 Hz, 1H), 5.16 (d, *J*=1.6 Hz, 1H), 4.31–4.35 (m, 1H), 4.12–4.15 (m, 1H), 4.00–4.03 (m, 1H), 3.88–3.91 (m, 1H), 0.96–1.05 (m, 28H).

*Step 6: Synthesis of O-((6aR,8S,9S,9aR)-8-(4-aminopyrrolo[2,1-f][1,2,4]triazin-7-yl)-2,2,4,4-tetra-isopropyltetrahydro-6H-furo[3,2-f][1,3,5,2,4]trioxadisilocin-9-yl) O-phenyl carbonothioate*

(6a*R*,8*S*,9*S*,9a*S*)-8-(4-aminopyrrolo[2,1-f][1,2,4]triazin-7-yl)-2,2,4,4-tetraisopropyl-tetrahydro-6H-furo[3,2-f][1,3,5,2,4]trioxadisilocin-9-ol (210 mg, 0.413 mmol) and DMAP (202 mg, 1.651 mmol) were dissolved in CH_2_Cl_2_ (10 mL) under Ar atmosphere. *O*-phenyl carbonochloridothioate (143 mg, 0.826 mmol) was added dropwise at 0 ºC. The reaction was stirred at 25 ºC under Ar atmosphere for 2 hours, and the resulting mixture was diluted with CH_2_Cl_2_ (100 mL), and then washed with water (3x 30 mL) and brine (30 mL). The organic layer was dried over anhydrous Na_2_SO_4_ and concentrated under reduced pressure. The resulting residue was purified by chromatography on SiO_2_ (isocratic elution with 3:1 EtOAc/petroleum ether) to provide the title compound (230 mg, 0.357 mmol, 86% yield). LRMS: (ES, m/z): 645.2 [M+H]. ^1^H NMR: (400 MHz, DMSO, ppm): δ 7.83 (s, 2H), 7.79 (s, 1H), 7.48 (t, *J*=8.0 Hz, 2H), 7.33 (t, *J*=7.2 Hz, 1H), 7.14 (d, *J*=7.6 Hz, 2H), 6.80 (d, *J*=4.4 Hz, 1H), 6.71 (d, *J*=4.4 Hz, 1H), 6.16 (dd, *J*=2.4, 5.6 Hz, 1H), 5.46 (d, *J*=2.0 Hz, 1H), 4.90 (dd, *J*=5.6, 8.8 Hz, 1H), 3.95–4.06 (m, 2H), 3.85–3.87 (m, 1H), 0.96–1.06 (m, 28H).

*Step 7: Synthesis of 7-((6aR,8R,9aS)-2,2,4,4-tetraisopropyltetrahydro-6H-furo[3,2-f][1,3,5,2,4]-trioxadisilocin-8-yl)pyrrolo[2,1-f][1,2,4]triazin-4-amine*

*O*-((6a*R*,8*S*,9*S*,9a*R*)-8-(4-aminopyrrolo[2,1-f][1,2,4]triazin-7-yl)-2,2,4,4-tetra-isopropyltetrahydro-6H-furo[3,2-f][1,3,5,2,4]trioxadisilocin-9-yl) *O*-phenyl carbonothioate (1.45 g, 2.248 mmol), AIBN (0.738 g, 4.50 mmol), and tributylstannane (3.93 g, 13.49 mmol) were dissolved in toluene (20 mL) under Ar atmosphere. The reaction was stirred at 80 ºC under Ar atmosphere for 3 hours, and the resulting solution was concentrated under reduced pressure. The resulting residue was purified by chromatography on SiO_2_ (isocratic elution with 2.5:1 EtOAc/petroleum ether) to provide the title compound (1.0 g, 2.029 mmol, 90% yield). LRMS: (ES, m/z): 493.4 [M+H]. ^1^H NMR: (400 MHz, DMSO, ppm): δ 7.81 (s, 1H), 7.79 (s, 2H), 6.81 (d, *J*=4.4 Hz, 1H), 6.60 (d, *J*=4.4 Hz, 1H), 5.46 (m, 1H), 4.58–4.62 (m, 1H), 3.90–4.00 (m, 1H), 3.75-3.80 (m, 2H), 2.20–2.30 (m, 1H), 2.30–2.40 (m, 1H), 0.98–1.06 (m, 28H).

*Step 8: Synthesis of (2R,3S,5R)-5-(4-aminopyrrolo[2,1-f][1,2,4]triazin-7-yl)-2-(hydroxymethyl)-tetrahydrofuran-3-ol*

7-((6a*R*,8*R*,9a*S*)-2,2,4,4-tetraisopropyltetrahydro-6H-furo[3,2-f][1,3,5,2,4]-trioxadisilocin-8-yl)pyrrolo-[2,1-f][1,2,4]triazin-4-amine (1.0 g, 2.029 mmol) was dissolved in THF (10 mL) under Ar atmosphere. A 1M THF solution of TBAF (0.531 g, 2.029 mmol) was added to the reaction solution dropwise at 20 ºC. After the resulting solution was stirred at 20 ºC under Ar atmosphere for 2 hours, the reaction mixture was concentrated under reduced pressure. The resulting residue was purified by chromatography on SiO_2_ (isocratic elution with 1:20 MeOH/CH_2_Cl_2_) to provide the title compound (300 mg, 1.199 mmol, 59.1% yield). LRMS: (ES, m/z): 251.2 [M+H]. ^1^H NMR: (400 MHz, DMSO, ppm): δ 7.83 (s, 1H), 7.81 (s, 2H), 6.84 (d, *J* = 4.8 Hz, 1H), 6.82 (d, *J*=4.4 Hz, 1H), 5.47-5.51 (m, 1H), 5.06 (d, *J*=4.0 Hz, 1H), 4.73 (t, *J*=6.0 Hz, 1H), 4.22 (m, 1H), 3.74–3.78 (m, 1H), 3.32–3.45 (m, 2H), 2.16–2.20 (m, 1H), 2.09–2.14 (m, 1H).

*Step 9: Synthesis of 7-((2R,4S,5R)-4-((tert-butyldimethylsilyl)oxy)-5-(((tert-butyldimethylsilyl)oxy)-methyl)tetrahydrofuran-2-yl)pyrrolo[2,1-f][1,2,4]triazin-4-amine*

(2*R*,3*S*,5*R*)-5-(4-aminopyrrolo[2,1-f][1,2,4]triazin-7-yl)-2-(hydroxymethyl)-tetrahydrofuran-3-ol

(300 mg, 1.199 mmol), imidazole (490 mg, 7.19 mmol), and *tert*-butylchlorodimethylsilane (723 mg, 4.80 mmol) were dissolved in DMF (4.0 mL) under Ar atmosphere. The reaction mixture was stirred at 20 ºC under Ar atmosphere for 5 hours, and the resulting mixture was diluted with EtOAc (100 mL), washed with water (3x 30 mL) and brine (30 mL). The organic layer was dried over anhydrous Na_2_SO_4_ and concentrated under reduced pressure. The resulting residue was purified by chromatography on SiO_2_ (isocratic elution with 2:1 EtOAc/petroleum ether) to provide the title compound (550 mg, 1.034 mmol, 86% yield). LRMS: (ES, m/z): 479.3 [M+H]. ^1^H NMR: (300 MHz, DMSO, ppm): δ 7.82 (s, 1H), 7.75 (s, 2H), 6.82 (d, *J*=4.5 Hz, 1H), 6.61 (d, *J*=4.5 Hz, 1H), 5.48–5.53 (m, 1H), 4.42 (m, 1H), 3.78 (m, 1H), 3.48–3.62 (m, 2H), 2.22–2.27 (m, 1H), 2.04–2.10 (m, 1H), 0.78–0.89 (m, 18H), 0.00–0.10 (m, 12H).

*Step 10: Synthesis of ((2R,3S,5R)-5-(4-aminopyrrolo[2,1-f][1,2,4]triazin-7-yl)-3-((tert-butyldimethyl-silyl)oxy)tetrahydrofuran-2-yl)methanol*

7-((2*R*,4*S*,5*R*)-4-((*tert*-butyldimethylsilyl)oxy)-5-(((*tert*-butyldimethylsilyl)oxy)-methyl)tetrahydrofuran-2-yl)pyrrolo[2,1-f][1,2,4]triazin-4-amine (500 mg, 1.044 mmol) was dissolved in THF (8.0 mL), H_2_O (2.0 mL) and TFA (2.0 mL) and stirred at 0 ºC for 4 hours. The reaction mixture was adjusted to a pH of 7.0 with NaHCO_3_ and then extracted with EtOAc (3x 30 mL) and washed with water (3x20 mL) and brine (20 mL). The organic layer was dried over anhydrous Na_2_SO_4_ and concentrated under reduced pressure. The resulting residue was purified by chromatography on SiO_2_ (isocratic elution with 2:1 EtOAc/petroleum ether) to provide the title compound (270 mg, 0.741 mmol, 70.9% yield). LRMS: (ES, m/z): 365.3 [M+H]. ^1^H NMR: (400 MHz, DMSO, ppm): δ 7.82 (s, 1H), 7.68 (s, 2H), 6.82 (d, *J*=4.4 Hz, 1H), 6.65 (d, *J*=4.4 Hz, 1H), 5.46–5.50 (m, 1H), 4.80 (t, *J*=5.6 Hz, 1H), 4.42 (d, *J*=4.2 Hz, 1H), 3.74–3.78 (m, 1H), 3.32–3.44 (m, 2H), 2.24–2.28 (m, 1H), 2.02–2.06 (m, 1H), 0.85–0.90 (m, 9H), 0.00–0.10 (m, 6H).

*Step 11: Synthesis of (2S,3S,5R)-5-(4-aminopyrrolo[2,1-f][1,2,4]triazin-7-yl)-3-((tert-butyldimethylsilyl)-oxy)tetrahydrofuran-2-carbaldehyde*

((2*R*,3*S*,5*R*)-5-(4-aminopyrrolo[2,1-f][1,2,4]triazin-7-yl)-3-((*tert*-butyldimethyl-silyl)oxy)tetrahydro-furan-2-yl)methanol (270 mg, 0.741 mmol) and IBX (270 mg, 0.963 mmol) were dissolved in MeCN (20.0 mL) under Ar atmosphere. The reaction mixture was stirred at 80 ºC under Ar atmosphere for 1 hour, the solid precipitation was filtered and the filtrate was concentrated under reduced pressure to provide the title compound (200 mg, 0.552 mmol, 74.5% yield), which was used in the subsequent step without further purification. LRMS: (ES, m/z): 363.2 [M+H]. ^1^H NMR: (400 MHz, DMSO, ppm): δ 9.59 (s, 1H), 7.84 (s, 1H), 7.73(s, 2H), 6.86 (d, *J*=4.4 Hz, 1H), 6.64 (d, *J*=4.4 Hz, 1H), 5.67–5.71 (m, 1H), 4.71 (d, *J*=4.4 Hz, 1H), 4.25 (s, 1H), 2.25–2.32 (m, 1H), 2.12–2.17 (m, 2H), 0.84–0.91 (m, 9H), 0.00–0.13 (m, 6H).

*Step 12: Synthesis of ((3S,5R)-5-(4-aminopyrrolo[2,1-f][1,2,4]triazin-7-yl)-3-((tert-butyldimethyl-silyl)oxy)tetrahydrofuran-2,2-diyl)dimethanol*

(2*S*,3*S*,5*R*)-5-(4-aminopyrrolo[2,1-f][1,2,4]triazin-7-yl)-3-((*tert*-butyldimethylsilyl)-oxy)tetrahydro-furan-2-carbaldehyde (200 mg, 0.552 mmol) was dissolved in 1,4-dioxane (10 mL) at 20 ºC. Next, formaldehyde (2.0 mL, 0.552 mmol) and 2N NaOH (2.0 mL, 4.00 mmol) were added. The resulting mixture was stirred at 20 ºC for 4 hours, and then adjusted to a pH of 7.0 with AcOH. The mixture was diluted with EtOAc (150 mL), and then washed with water (3x 30 mL) and brine (30 mL). The organic layer was dried over anhydrous Na_2_SO_4_ and concentrated under reduced pressure. The resulting residue was dissolved in EtOH (10 mL) at 0 ºC, and NaBH_4_ (41.7 mg, 1.103 mmol) was then added at 0 ºC. The resulting mixture was stirred at 20 ºC for 16 hours, and then adjusted to a pH of 7.0 with AcOH. The mixture was extracted with EtOAc (150 mL), and then washed with water (3x 30 mL) and brine (30 mL). The organic layer was dried over anhydrous Na_2_SO_4_ and concentrated under reduced pressure. The resulting residue was purified by chromatography on SiO_2_ (isocratic elution with 1:10 MeOH/CH_2_Cl_2_) to provide the title compound (60 mg, 0.152 mmol, 27.6% yield). LRMS: (ES, m/z): 395.2 [M+H]. ^1^H NMR: (400 MHz, DMSO, ppm): δ 7.82 (s, 1H), 7.70 (s, 2H), 6.82 (d, *J*=4.4 Hz, 1H), 6.66 (d, *J*=4.4 Hz, 1H), 5.50-5.60 (m, 1H), 4.68 (m, 1H), 4.52 (d, *J*=4.4 Hz, 1H), 4.22 (m, 1H), 3.53–3.60 (m, 2H), 2.35–2.45 (m, 1H), 2.00–2.10 (m, 1H), 0.90 (s, 9H), 0.08–0.09 (m, 6H).

*Step 13: Synthesis of (2R,3S,5R)-5-(4-aminopyrrolo[2,1-f][1,2,4]triazin-7-yl)-3-((tert-butyldimethylsilyl)-oxy)-2-(hydroxymethyl)tetrahydrofuran-2-carbaldehyde*

((3*S*,5*R*)-5-(4-aminopyrrolo[2,1-f][1,2,4]triazin-7-yl)-3-((*tert*-butyldimethyl-silyl)oxy)tetrahydrofuran-2,2-diyl)dimethanol (90 mg, 0.228 mmol) and IBX (256 mg, 0.912 mmol) were dissolved in MeCN (15.0 mL) under Ar atmosphere. The reaction mixture was stirred at 30 ºC under Ar atmosphere for 48 hours, after which the solid precipitate was filtered. The filtrate was concentrated under reduced pressure to provide the title compound (7 mg, 0.018 mmol, 70.4% yield), which was used in the subsequent step without further purification.

*Step 14: Synthesis of ((2R,3S,5R)-5-(4-aminopyrrolo[2,1-f][1,2,4]triazin-7-yl)-3-((tert-butyldimethyl-silyl)oxy)-2-ethynyltetrahydrofuran-2-yl)methanol*

(2*R*,3*S*,5*R*)-5-(4-aminopyrrolo[2,1-f][1,2,4]triazin-7-yl)-3-((*tert*-butyldimethylsilyl)-oxy)-2-(hydroxymethyl)tetrahydrofuran-2-carbaldehyde (80 mg, 0.204 mmol) and K_2_CO_3_ (85 mg, 0.611 mmol) were dissolved in MeOH (10 mL) under Ar atmosphere. Dimethyl-(1-diazo-2-oxopropyl)phosphonate (78 mg, 0.408 mmol) was added to the reaction mixture at 0 ºC dropwise as a solution in MeOH. The reaction mixture was stirred at 25 ºC under Ar atmosphere for 16 hours, and then filtrated and concentrated under reduced pressure. The resulting residue was purified by chromatography on SiO_2_ (isocratic elution with 1:10 MeOH/CH_2_Cl_2_) to provide the title compound (50 mg, 0.129 mmol, 63.1% yield). LRMS: (ES, m/z): 389.3 [M+H].

*Step 15: Synthesis of (2R,3S,5R)-5-(4-aminopyrrolo[2,1-f][1,2,4]triazin-7-yl)-2-ethynyl-2-(hydroxymethyl)-tetrahydrofuran-3-ol* **(Compound 16)**

((2*R*,3*S*,5*R*)-5-(4-aminopyrrolo[2,1-f][1,2,4]triazin-7-yl)-3-((*tert*-butyldimethyl-silyl)oxy)-2-ethynyltetrahydrofuran-2-yl)methanol (50 mg, 0.129 mmol) and a 1M THF solution of TBAF (33.6 mg, 0.129 mL, 0.129 mmol) were combined in THF (5.0 mL) under Ar atmosphere. The resulting solution was stirred at 25 ºC under Ar atmosphere for 2 hours, and the reaction mixture concentrated under reduced pressure. The resulting residue was then purified by preparative HPLC [Prep-HPLC-011(Waters); Column: Xbridge Prep C18 Column, 19*150mm; Mobile phase: water (with 10 mmol NH_4_HCO_3_) and MeCN; Detector: UV 254 and 220 nm] to provide the title compound (25 mg, 0.091 mmol, 70.8% yield). LRMS: (ES, m/z): 275.1 [M+H]. ^1^H NMR: (400 MHz, DMSO, ppm): δ 7.82 (s, 1H), 7.69 (s, 2H), 6.82 (d, *J*=4.4 Hz, 1H), 6.69 (d, *J*=4.0 Hz, 1H), 5.60 (t, *J*=7.6 Hz, 1H), 5.28 (d, *J*=4.2 Hz,1H), 5.06 (d, *J*=6.8 Hz, 1H ), 4.30–4.34 (m, 1H), 3.45–3.56 (m, 2H), 3.38 (s, 1H), 2.30–2.37 (m, 1H), 2.17–2.13 (m, 1H).

**Synthesis of Compound 17** *rac* *4-(6-amino-2-fluoro-9H-purin-9-yl)-2-ethynyl-2-(hydroxy-methyl)cyclopentan-1-ol* **(Compound 17)**

Compound 17 was prepared according to the experimental procedure described in: Alexandre, F.-R., *et al*. *J. Med. Chem.* **2018**, *61*, 9218. [2]

LRMS: (ES, m/z): 292.40 [M+H]^+^. ^1^H NMR: (400 MHz, *d_6_*-DMSO, *ppm*): δ 8.21 (s, 1H), 7.77 (brs, 2H), 5.09-5.01 (m, 3H), 4.33 (t, *J*=7.61 Hz, 1H), 3.56–3.50 (m, 2H), 3.05 (s, 1H), 2.30–2.14 (m, 4H). ^19^F NMR: (376 MHz, *d_6_*-DMSO, ppm): δ 52.10 (s, 1F).

**Synthesis of Compound 18** *rac* *4-(6-amino-2-chloro-9H-purin-9-yl)-2-ethynyl-2-(hydroxy-methyl)-cyclopentan-1-ol* **(Compound 18)**

Compound 18 was prepared according to the experimental procedure described in: Alexandre, F.-R., *et al*. *J. Med. Chem.* **2018**, *61*, 9218. [2]

LRMS: (ES, m/z): 308.30 [M+H]^+^. ^1^H NMR: (400 MHz, *d_6_*-DMSO, ppm): δ 8.26 (s, 1H), 7.76 (brs, 2H), 5.12–5.04 (m, 3H), 4.35–4.29 (m, 1H), 3.57-3.53 (m, 2H), 3.05 (s, 1H), 2.29–2.13 (m, 4H).

**Synthesis of Compound 19** *rac (1S,2S,4R)-4-(4-amino-2-chloro-7H-pyrrolo[2,3-d]pyrimidin-7-yl)-2-ethynyl-2-(hydroxymethyl)cyclopentan-1-ol* **(Compound 19)**

Compound 19 was prepared according to the experimental procedure described in: Alexandre, F.-R., *et al*. *J. Med. Chem.* 2018, *61*, 9218. [2]

LRMS: (ES, m/z): 307.30 [M+H]^+^. ^1^H NMR: (500 MHz, *d_6_*-DMSO): δ 7.45 (s, 2H), 7.24 (d, *J*=3.6 Hz, 1H), 6.57 (d, *J*=3.6 Hz, 1H), 5.28–5.16 (m, 1H), 5.03 (d, *J*=5.2 Hz, 1H), 4.98 (d, *J*=5.4 Hz, 1H), 4.26 (q, *J*=7.5 Hz, 1H), 3.52 (qd, *J*=10.8, 5.0 Hz, 2H), 3.03 (s, 1H), 2.18 (dt, *J*=12.8, 8.2 Hz, 2H), 2.11–2.01 (m, 2H).

**Synthesis of Compound 20** *2-amino-9-((1R,3S,4S)-3-ethynyl-4-hydroxy-3-(hydroxymethyl)-cyclopentyl)-1,9-dihydro-6H-purin-6-one* **(Compound 20)**

Compound 20 was prepared according to the experimental procedure described in: Alexandre, F.-R., *et al*. *J. Med. Chem.* **2018**, *61*, 9218. [2]

LRMS: (ES, m/z): 290.40 [M+H]^+^. ^1^H NMR: (400 MHz, *d_6_*-DMSO, ppm): δ 10.55 (s, 1H), 7.81 (s, 1H), 6.44 (brs, 2H), 5.01 (t, *J*=5.82 Hz, 1H), 4.98–4.91 (m, 2H), 4.29–4.24 (m, 1H), 3.56–3.48 (m, 2H), 3.04 (s, 1H), 2.22-2.11 (m, 3H), 2.08–2.02 (m, 1H).

**References**

1. McLaughlin M, Kong J, Belyk KM, Chen B, Gibson AW, Keen SP, et al. Enantioselective Synthesis of 4'-Ethynyl-2-fluoro-2'-deoxyadenosine (EFdA) via Enzymatic Desymmetrization. Org Lett. 2017;19(4):926-929. doi: 10.1021/acs.orglett.7b00091. PMID: 28165251.

2. Alexandre FR, Rahali R, Rahali H, Guillon S, Convard T, Fillgrove K, et al. Synthesis and Antiviral Evaluation of Carbocyclic Nucleoside Analogs of Nucleoside Reverse Transcriptase Translocation Inhibitor MK-8591 (4'-Ethynyl-2-fluoro-2'-deoxyadenosine). J Med Chem. 2018;61(20):9218-9228. doi: 10.1021/acs.jmedchem.8b00141. PMID: 30265808.
