# Supplementary material for: MK-8527 is a novel inhibitor of HIV-1 reverse transcriptase translocation with potential for extended-duration dosing
Source: PLoS Biol. 2025 Aug 26;23(8):e3003308. doi: 10.1371/journal.pbio.3003308 (PMC12380353; doi:10.1371/journal.pbio.3003308)
Supplement: S1 Table — Statistics for the highest resolution shell are shown in parentheses. (DOCX) [file pbio.3003308.s002.docx]

## S1 Table. Data collection and refinement statistics.

|  | HIVRT ternary complex with MK-8527-TP |
| --- | --- |
| Wavelength (Å) | 1.00 |
| Resolution range | 85.41 - 2.73 (3.07 - 2.73) |
| Space group | C 2 2 21 |
| Unit cell | 163.91 170.81 101.45 90 90 90 |
| Total reflections | 157371 |
| Unique reflections | 23212 (1161) |
| Multiplicity | 6.8 |
| Completeness, spherical/ellipsoidal (%) | 61.0 (10.4)/93.4 (73.3) |
| Mean I/sigma(I) | 6.2 (1.5) |
| Wilson B-factor | 100.26 |
| R-merge | 0.138 (1.150) |
| R-meas | 0.150 (1.245) |
| R-pim | 0.057 (0.474) |
| CC1/2 | 0.996 (0.665) |
| Reflections used in refinement | 23188 |
| R-work | 0.2014 |
| R-free | 0.2284 |
| Number of non-hydrogen atoms | 8622 |
| macromolecules | 8587 |
| ligands | 35 |
| solvent | 0 |
| Protein residues | 944 |
| RMS(bonds) | 0.011 |
| RMS(angles) | 1.21 |
| Ramachandran favored (%) | 91.54 |
| Ramachandran allowed (%) | 7.60 |
| Ramachandran outliers (%) | 0.86 |
| Rotamer outliers (%) | 8.36 |
| Clashscore | 10.40 |
| Average B-factor | 55.49 |
| macromolecules | 54.88 |
| ligands | 47.28 |

Statistics for the highest-resolution shell are shown in parentheses.
