# Supplementary figures and images for: MK-8527 is a novel inhibitor of HIV-1 reverse transcriptase translocation with potential for extended-duration dosing
Source: PLoS Biol. 2025 Aug 26;23(8):e3003308. doi: 10.1371/journal.pbio.3003308 (PMC12380353; doi:10.1371/journal.pbio.3003308)

Figure 7B

5 uM ddATP present in all

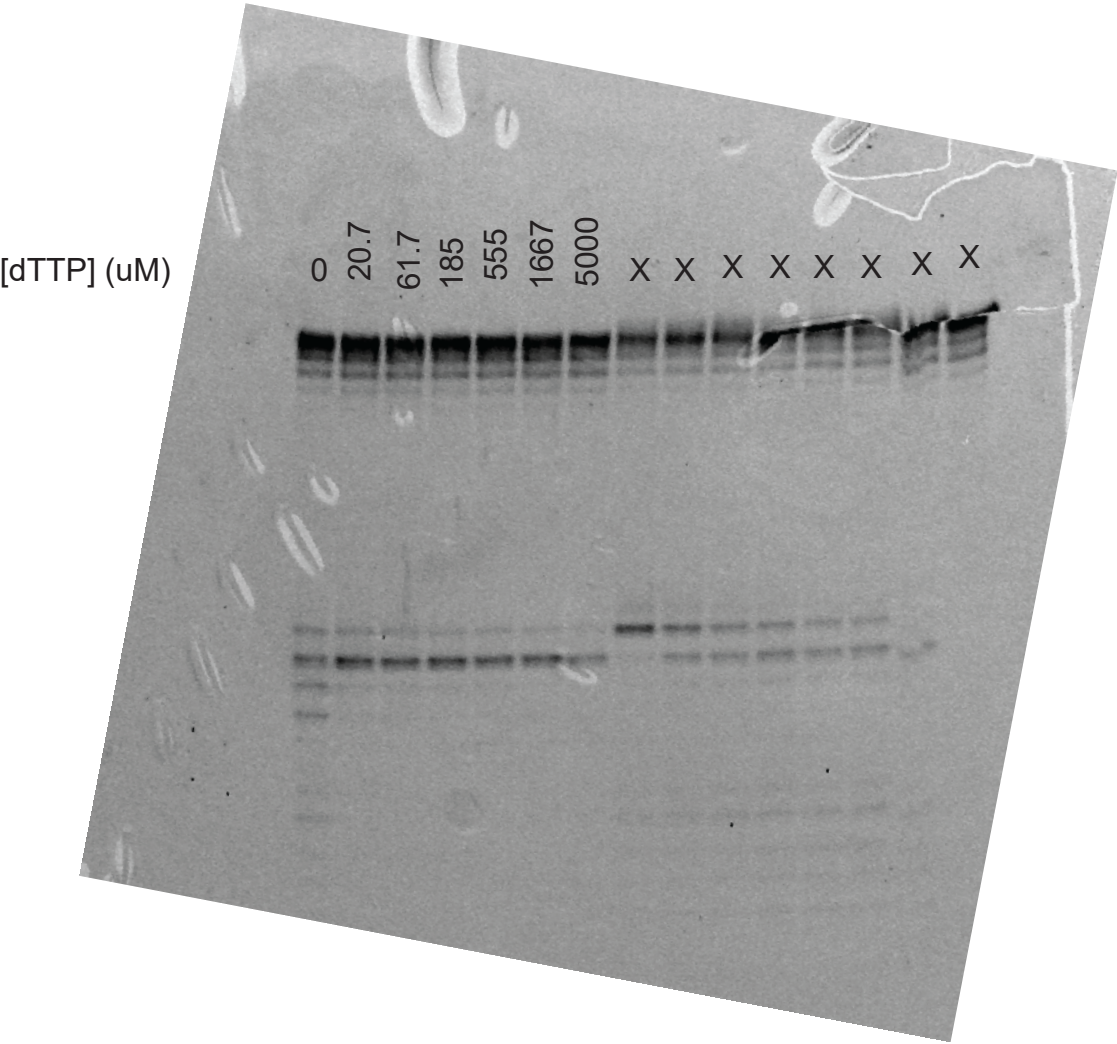

**Figure 7C**

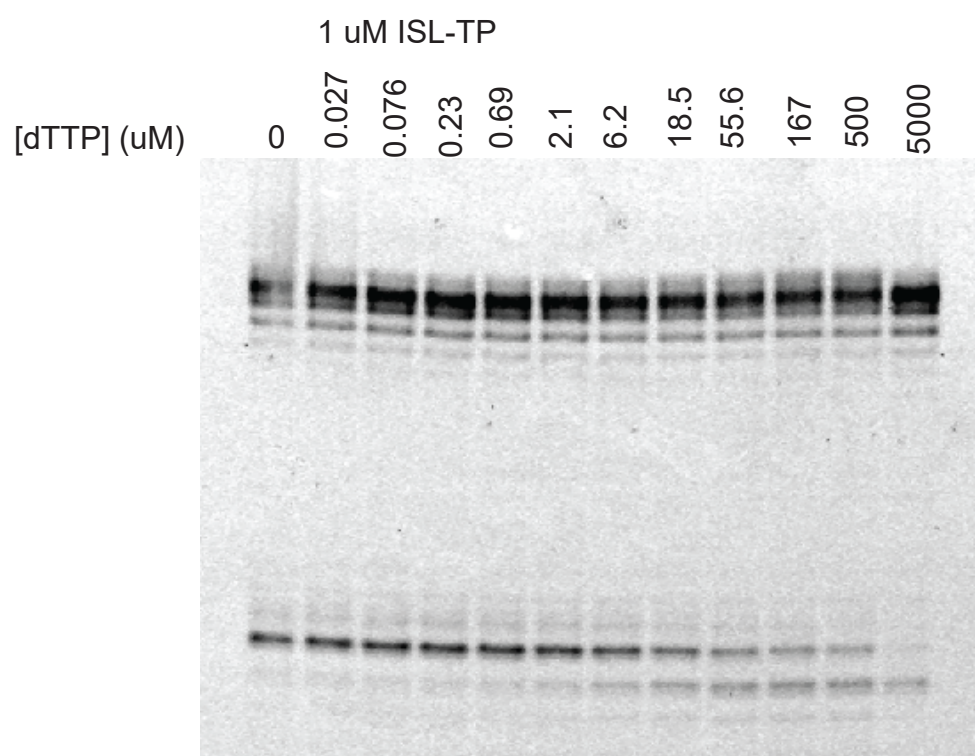

**Figure 7D**

|             |   |   |   |       |      |      |     |     |      |      |     |     |       |
|-------------|---|---|---|-------|------|------|-----|-----|------|------|-----|-----|-------|
| ISL-TP (uM) |   |   | 1 | 1     | 1    | 1    | 1   | 1   | 1    | 1    | 1   | 1   | 1     |
| [dTTP] (uM) | X | X | 0 | 0.076 | 0.23 | 0.69 | 2.1 | 6.2 | 18.5 | 55.6 | 167 | 500 | 10000 |

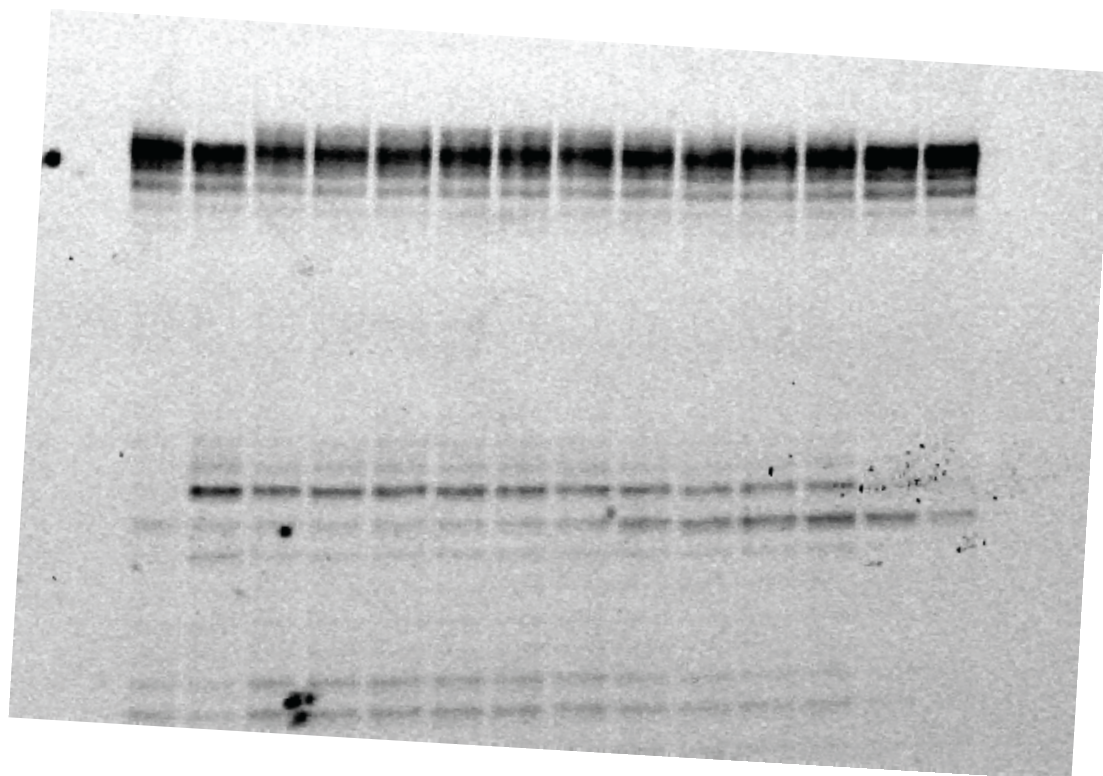

Supplement: S1 Raw Images — Iron footprinting assay to evaluate the position of RT on the primer and template. ddATP, dideoxyadenosine triphosphate; dTTP, deoxythymidine triphosphate; ISL, islatravir; RT, reverse transcriptase; TP, triphosphate. (PDF) [file pbio.3003308.s003.pdf]

Figure 8B

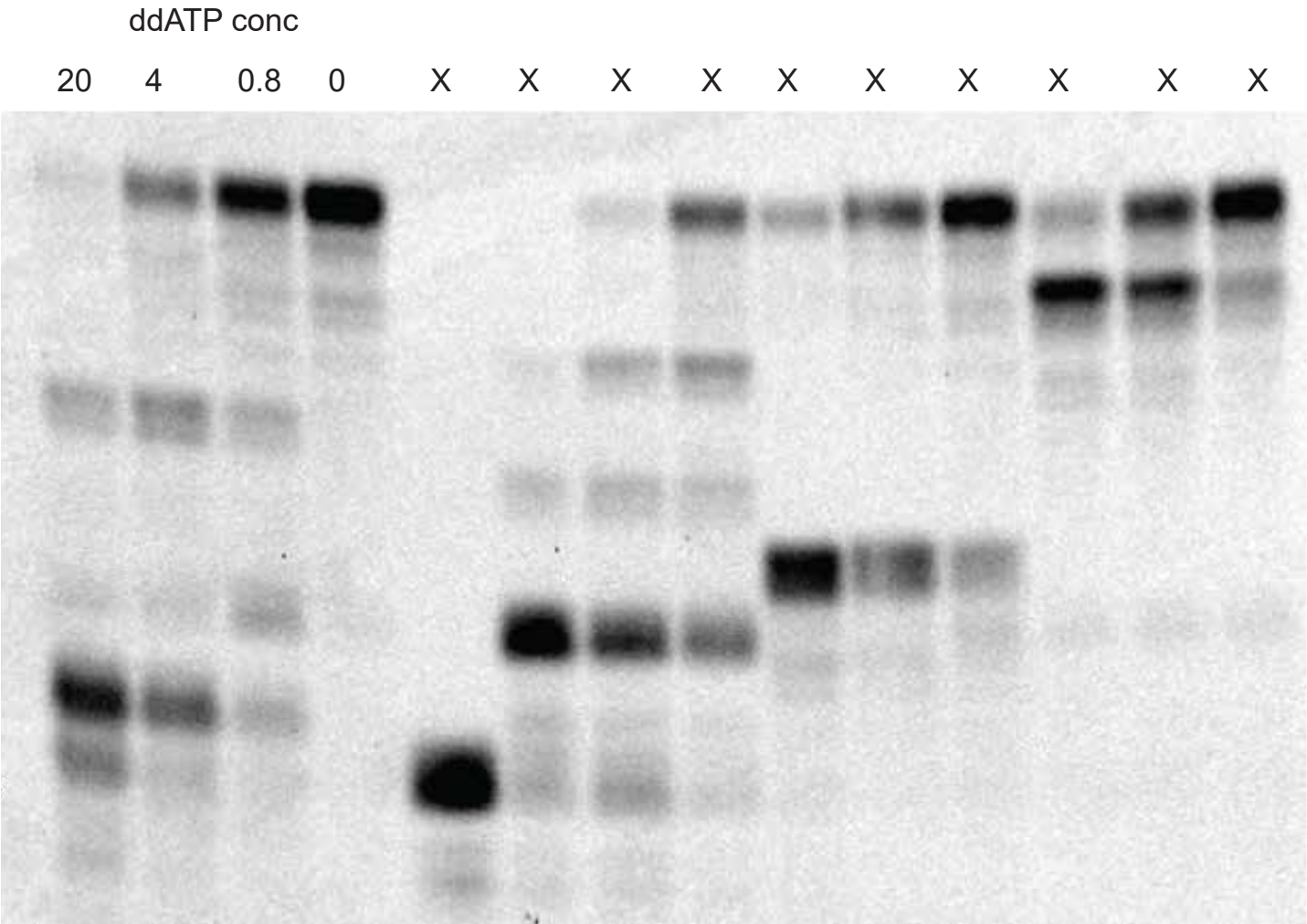

Figure 8C and 8D

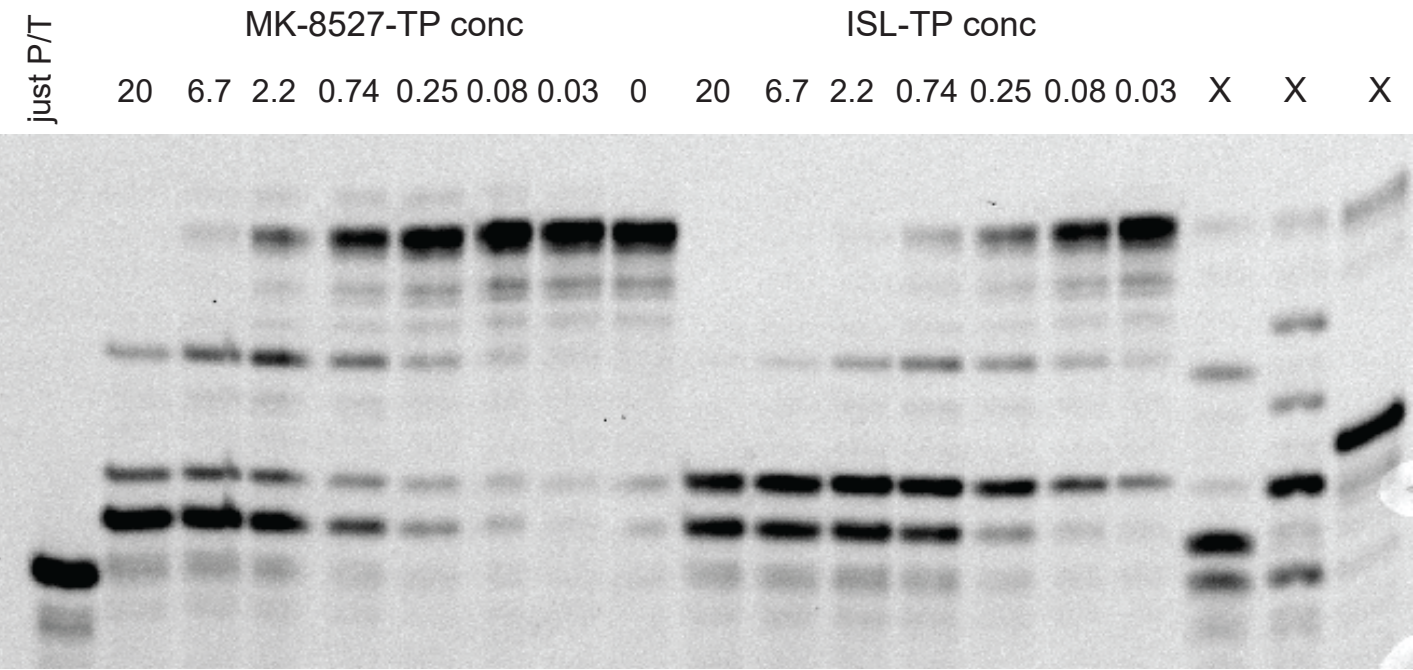

Supplement: S2 Raw Images — HIV-RT primer extension assay to evaluate the chain termination mechanism of MK-8527-TP compared with ISL-TP and ddATP (an NRTI). ddATP, dideoxyadenosine triphosphate; dTTP, deoxythymidine triphosphate; ISL, islatravir; NRTI, nucleos(t)ide reverse transcriptase inhibitor; RT, reverse transcriptase; TP, triphosphate. (PDF) [file pbio.3003308.s004.pdf]
